# A novel lymphatic pattern promotes metastasis of cervical cancer in a hypoxic tumour-associated macrophage-dependent manner

**DOI:** 10.1007/s10456-020-09766-2

**Published:** 2021-01-23

**Authors:** Xiao-Jing Chen, Wen-Fei Wei, Zi-Ci Wang, Nisha Wang, Chu-Hong Guo, Chen-Fei Zhou, Luo-Jiao Liang, Sha Wu, Li Liang, Wei Wang

**Affiliations:** 1 Department of Obstetrics and Gynecology, The First Affiliated Hospital of Guangzhou Medical University, 151 Yanjiang Road, Yuexiu District, Guangzhou, 510120 People’s Republic of China; 2 Department of Immunology/Guangdong Provincial Key Laboratory of Proteomics, School of Basic Medical Sciences, Southern Medical University, 1838 Guangzhou Avenue North, Baiyun District, Guangzhou, 510515 People’s Republic of China; 3 Department of Pathology, Nanfang Hospital, Southern Medical University, 1838 Guangzhou Avenue North, Baiyun District, Guangzhou, 510515 People’s Republic of China; 4 Department of Biochemistry and Molecular Biology, School of Basic Medical Sciences, Southern Medical University, 1838 Guangzhou Avenue North, Baiyun District, Guangzhou, 510515 People’s Republic of China

**Keywords:** Hypoxia, Lymph node metastasis, Lymphangiogenesis, Tumour-associated macrophages, Lymphatic vessels encapsulated by tumour-associated macrophages (LVEM)

## Abstract

Lymphatic remodelling in the hypoxic tumour microenvironment (TME) is critically involved in the metastasis of cervical squamous cell carcinoma (CSCC); however, its underlying mechanisms remain unclear. Here, we uncovered a novel lymphatic pattern in the hypoxic TME, wherein lymphatic vessels (LVs) are encapsulated by tumour-associated macrophages (TAMs) to form an interconnected network. We describe these aggregates as LVEM (LVs encapsulated by TAMs) considering their advantageous metastatic capacity and active involvement in early lymph node metastasis (LNM). Mechanistic investigations revealed that interleukin-10 (IL-10) derived from hypoxic TAMs adjacent to LVs was a prerequisite for lymphangiogenesis and LVEM formation through its induction of Sp1 upregulation in lymphatic endothelial cells (LECs). Interestingly, Sp1^high^ LECs promoted the transactivation of C–C motif chemokine ligand 1 (CCL1) to facilitate TAM and tumour cell recruitment, thereby forming a positive feedback loop to strengthen the LVEM formation. Knockdown of Sp1 or blockage of CCL1 abrogated LVEM and consequently attenuated LNM. Notably, CSCC^non-LNM^ is largely devoid of hypoxic TAMs and the resultant LVEM, which might explain its metastatic delay. These findings identify a novel and efficient metastasis-promoting lymphatic pattern in the hypoxic TME, which might provide new targets for anti-metastasis therapy and prognostic assessment.

**Supplementary Information:**

The online version contains supplementary material available at 10.1007/s10456-020-09766-2.

## Introduction

Hypoxia, a distinguished feature of solid tumours, often leads to the development of localized tumour heterogeneous environments [1]. Hypoxia-induced tumour heterogeneity, which promotes metastatic progression, is a major challenge in the clinical management of malignancy [2]. Although the significant prognostic effects of hypoxia have been elucidated, the mechanisms mediating this deadly biological process relative to lymph node metastasis (LNM) remain poorly understood. In most cases, tumour cells must escape the primary tumour site, translocate to the lymphatic vessels (LVs), survive in the circulation, and subsequently grow at a new site to establish LNM [3]. Migration of tumour cells into lymphatic circulation and entry into lymph nodes (LNs) are greatly facilitated by tumour lymphangiogenesis, a process that generates neo-lymphatics from pre-existing conduits [4]. Previous studies emphasize that neo-lymphatics within the hypoxic tumour microenvironment (TME) serve as a highway for tumour cell dissemination from their primary site to regional LNs [5]. However, little attention has been devoted to analysing their properties and composition in the hypoxic niches. It is necessary to understand the characteristics of these metastasis-associated LVs and identify specific markers for their definition.

The remodelling of regional LVs to facilitate tumour cell dissemination plays an important pro-metastatic role in solid tumours, especially in cervical squamous cell carcinoma (CSCC) [6]. However, recent studies suggest that LVs are heterogeneous in their differential response to various micro-environmental signals and thus display distinct phenotypes and functions, which hinder their application in diagnosis and targeted therapy [7]. Different LV populations that secrete distinct profiles of cytokines have been identified in a variety of cancers [8]. Similar to tumour cells, the maintenance of metastasis-associated LV properties requires a supportive niche [9]. Macrophages, the most important component of inflammatory infiltrates in the TME, contain different subpopulations, which primarily depend on their localization within the tumour [10]. Hypoxia shapes and induces specific macrophage phenotypes that serve tumour malignancy [11]. In our previous study, macrophages in hypoxic tumour niches differentiated into tumour-associated macrophages (TAMs) with an immunosuppressive phenotype, and their presence in high numbers is strongly associated with LNM of CSCC [12], suggesting their novel roles in supporting the metastatic capacity of LVs. Interestingly, we found a subset of TAMs adjacent to LVs in the hypoxic TME, with hypoxic TAMs more proximal to the LVs than normoxic TAMs. Further mechanistic studies revealed that these hypoxic TAMs might orchestrate cytokine and chemokine networks in the TME and thereby potentially influence cancer progression and metastasis [13]. However, the cytokine profile of TAMs in hypoxic milieu remains largely unknown, and it is unclear whether there exist exclusively hypoxic TAM-derived cytokines that are functionally essential to lymphatic remodelling. Hypoxia activates lymphatic endothelial cells (LECs) to release inflammatory mediators and increase the expression of specific oncogenes [14]. Additionally, hypoxia induces functional responses in LECs to promote metastatic progression [15]. These findings suggest that a certain lymphatic subtype in the hypoxic TME might profoundly affect LNM of CSCC; however, it remains unclear how this lymphatic subtype is established in CSCC and, importantly, whether and how it promotes lymphatic metastasis.

In this study, we show that in addition to classically described LVs, there exists a special type of activated LV that forms a cross-talk network with the hypoxic TME. This pattern of LVs encapsulated by TAMs (LVEM) is prevalent in hypoxic regions of CSCC and induced by a subset of TAMs adjacent to LVs. Importantly, abolishing the LVEM suppresses lymphatic metastasis in vivo. These data suggest that the LVEM might represent a novel mechanism for hypoxic TAM-dependent lymphatic metastasis and a new target for the prevention and treatment of CSCC metastasis.

## Materials and methods

### Cell lines and culture

Human CSCC cell lines (SiHa) and the human monocytic cell line THP-1 were purchased from American Type Culture Collection (ATCC, Manassas, VA, USA) and cultured according to their guidelines_._ Human dermal LECs (HDLECs) were purchased from ScienCell and cultured in endothelial cell medium (ScienCell, Carlsbad, CA, USA) supplemented with 10% foetal bovine serum and endothelial growth medium supplements (ScienCell). Murine TC-1 cells derived from primary epithelial cells of C57BL/6 mice co-transformed with HPV-16 E6/E7 and activation of Ras oncogenes and RAW264.7 macrophages were obtained from China Centre for Type Culture Collection (CCTCC) and cultured according to their guidelines. All cell lines were routinely checked for mycoplasma contamination (Lonza, Morrisville, NC, USA) and authenticated by their source organizations before purchase. Hypoxia treatment was administered by placing the cells in a hypoxic incubator (Thermo Fisher Scientific, Waltham, MA, USA) maintained at low oxygen tension (1% O_2_, 5% CO_2_, and 94% N_2_). Normoxia treatment was administered by placing the cells in a normoxic incubator (21% O_2_, 5% CO_2_, and 74% N_2_).

### Clinical specimens

A total of 75 archived, formalin-fixed, and paraffin-embedded CSCC specimens were obtained from the Department of Gynecological Oncology of The First Affiliated Hospital of Guangzhou Medical University between 2013 and 2014. They are all primary tumour samples, which were taken from CSCC patients that underwent abdominal radical hysterectomy without prior radiotherapy and chemotherapy. All the tissue were subjected to immunohistochemical and immunofluorescence analyses in this study. The study was approved by the Institutional Research Ethics Committee of The First Affiliated Hospital of Guangzhou Medical University. Details of the clinicopathologic characteristics are provided in Table 1. Each section was evaluated by two experienced pathologists who were blinded to patient clinicopathological data.

**Table 1:** Association of the clinicopathologic variables with the expression levels of CAIX, LV and LVEM

Clinicopathological indexes	No. of patients	CAIX			LV			LVEM		
		Low	High	*P*	Low	High	*P*	Low	High	*P*
Age (years)				0.877			0.757			0.662
< 45	35	19	16		17	18		14	21	
≥ 45	40	21	19		18	22		18	22	
FIGO stage				0.038*			0.040*			0.011*
I–IIA	48	28	20		26	22		27	21	
IIB–IV	27	9	18		8	19		7	20	
Histology				0.597			0.749			0.837
High	20	10	10		9	11		9	11	
Middle	32	19	13		16	16		17	15	
Low	23	15	8		13	10		12	11	
LNM				0.024*			0.029*			0.008*
No	45	27	18		25	20		26	19	
Yes	30	10	20		9	21		8	22	
Parametrial involvement										
No	60	27	33	0.563	30	30	0.817	30	30	0.488
Yes	15	8	7		8	7		6	9	
LVSI										
No	55	34	21	0.015*	35	20	0.067	38	17	0.002*
Yes	20	6	14		8	12		6	14	

*LV* lymphatic vessel, *LVEM* lymphatic vessels encapsulated by tumour-associated macrophages, *LNM* lymph node metastasis, *LVSI* lymphovascular space invasion

^*^Statistical significance (*P* < 0.05) was calculated using the Chi-squared test

### Conditioned medium (CM) preparation

Our previous studies confirmed that hypoxic TME is a crucial factor in inducing the phenotype and functional transformation of macrophages [12]. It is reported that most of the cytokines between mouse and human are highly conserved. The vast majority of their interactions are conserved [16]. In this way, RAW264.7 macrophages with culture medium under normoxia were referred to as “M0-N”, where those cultured under hypoxia were referred to as “M0-H”. “TAM-N” were obtained by culturing RAW264.7 macrophages with SiHa CM under normoxia, and “TAM-H” were obtained by culturing RAW264.7 macrophages with SiHa CM under hypoxia.

Cells were grown to ~ 80% confluence in different CM. After washing in fresh medium, cells were incubated for 48 h in serum-free fresh medium at 37 °C. CM was then harvested, centrifuged at 2000×*g* for 5 min, filtered using a 0.2-μm membrane syringe filter (Millipore, Billerica, MA, USA) to eliminate cell debris, and stored at − 80 °C until further study.

### Cytokine array

Assessment of cytokines secreted by macrophages was performed using a cytokine antibody array (mouse cytokine antibody array GSM-CAA-4000; RayBiotech, Peachtree Corners, GA, USA) that detects 200 cytokines. Medium from ~ 80% confluent macrophages (M0-N-1, M0-N-2, M0-N-3, M0-H-1, M0-H-2, M0-H-3, TAM-N-1, TAM-N-2, TAM-N-3, TAM-H-1, TAM-H-2, and TAM-H-3, respectively) were replaced with serum-free medium, which was harvested after 48 h, followed by removal of particulates by brief centrifugation, aliquoting, and freezing. Fresh aliquots were quantified, and 100 μl was used for the assay according to manufacturer instructions. Results were analysed using the RayBiotech Analyzer program, which normalized the signals using internal positive and negative controls included on the array.

### Popliteal lymphatic metastasis model

Female C57BL/6 mice (6–8 weeks old, 20–23 g) were purchased from the Experimental Animal Centre, Guangzhou Medical University (Guangzhou, P.R. China). All experimental procedures were approved by the Institutional Animal Care and Use Committee of Guangzhou Medical University. The popliteal lymphatic metastasis model was established in C57BL/6 mice by inoculating the footpad with TC-1 cells (5 × 10^6^; HPV-16 E6/E7-positive TC-1 mouse tumour model, which was used as the cervical cancer mouse model [17, 18]). Tumour size (mm^3^) was measured three times weekly and calculated using the following formula: volume = (width)^2^ × length/2. When footpad tumour size reached 50 mm^3^, macrophage supernatants from different treatment conditions (10 μl) were then injected into the centre of the tumours (*n* = 5/group, repeated twice) daily for 2 weeks. After 2 weeks of induction, primary tumours reached a comparable size of ~ 150 mm^3^, at which time the footpad tumours and popliteal LNs were collected for study. Positive LNs were identified by staining for the epithelial marker cytokeratin 7 (CK7; #17,513–1-AP; Proteintech) and detecting CK7-positivity using a Nikon upright microscope (Nikon, Tokyo, Japan). The ratio of metastasis-positive to total dissected popliteal LNs was calculated.

### In vivo TAM depletion model

A TAM depletion model was established in female C57BL/6 mice by inoculating the footpad with TC-1 cells (5 × 10^6^). When footpad tumour size reached 50 mm^3^, clodronate liposomes (CLs) and control liposomes (1 mg/mouse) were then multipoint injected into the tumours (*n* = 5/group, repeated twice) daily for 2 weeks. When the primary tumours reached a comparable size of ~ 150 mm^3^, the footpad tumours and popliteal LNs were collected for study.

### Establishment of Sp1-silenced and overexpressed stable lines

Lenti-GFP vectors containing an *Sp1* overexpression sequence, *Sp1*-knockdown sequence (sh-Sp1), and their respective negative control sequences (mock and scramble) were all purchased from GeneChem Inc. (Shanghai, P.R. China). HDLECs were transfected with lenti-GFP/Sp1, lenti-GFP/sh-Sp1, and their respective control lentiviral vector according to the manufacturer’s instructions. Lentivirus-mediated Sp1-silenced and Sp1-overexpressed stable cells were selected by puromycin (2 μg/ml).

### Cell transfection

Cells were transfected with 10 µM CCR8 siRNA (si-CCR8) or a negative control siRNA (si-NC) (GenePharma, P.R. China) using Lipofectamine™ 2000 (Invitrogen, USA) according to the manufacturer's instructions. HDLECs were also transfected with 4 μg plasmids (pcDNA3.1-Sp1/pcDNA3.1-NC and pGL3-CCL1-promoter) with Lipofectamine™ 2000 the same way as siRNA. The gene silencing efficiency of transfected cells was more than 90%, as confirmed by the detection of RT-qPCR.

### Tube formation in vitro

HDLECs tube formation assay in vitro was performed as described in our previous study [19].

### In vivo tube formation

HDLECs with different conditions (5 × 10^6^) and M2-polarized THP-1 macrophages (5 × 10^6^) were mixed at a ratio of 1:1. Matrigel (BD Biosciences, San Jose, CA, USA) and the cell mixture were combined at a ratio of 3:2, respectively, and 50-µl of the mixture was injected subcutaneously into female nude mice (6-weeks old). After 10 days of induction, the mice were euthanized. The Matrigel plug was paraffin-embedded and analysed by haematoxylin- eosin staining. Tube morphogenesis was assessed by phase-contrast microscopy (LSM 880 with Airyscan, Germany), and tube formation was quantified by counting the number of cells in branch-point capillaries (≥ 3 cells/branch) in three random fields using ImageJ software (National Institutes of Health, Bethesda, MD, USA).

### Western blot

Total proteins were extracted using radioimmunoprecipitation assay extraction reagents (Solarbio, Beijing, P.R. China). A total of 50 μg of protein was separated by 10% sodium dodecyl sulphate polyacrylamide gel electrophoresis and subsequently transferred to polyvinylidene difluoride membranes. The membranes were blocked with 5% bovine serum albumin for 1 h at 26 °C, incubated with the appropriate primary antibody at 4 °C overnight, and subsequently incubated with horseradish peroxidase-labelled secondary antibody for 1 h at 26 °C before detection by enhanced chemiluminescence (Thermo Fisher Scientific, USA). β-actin was used as an internal control for protein loading and analysis. The primary antibodies include anti-Sp1 (#sc-420; applied at 1:100; Santa Cruz Biotechnology), anti-signal transducer and activator of transcription 3 (STAT3; #D1A5; applied at 1:1000; CST), and anti-phosphorylated (p)-STAT3 (#D3A7; applied at 1:1000; CST). The secondary antibodies were horseradish peroxidase-conjugated anti-rabbit and -mouse immunoglobulin-G antibodies (both applied at 1:5000; Abcam, Cambridge, UK).

### Quantitative reverse transcription-polymerase chain reaction (qRT-PCR)

Total RNA was extracted from cells by Trizol Reagent (Solarbio, Beijing, P.R. China). For the mRNA analysis, complementary DNA (cDNA) was randomly primed from 1.0 μg of total RNA using the Prime Script (R) RT reagent kit (TaKaRa). Real-time PCR was subsequently performed in triplicate with a 1:4 dilution of cDNA using the SYBR Premix Ex Taq kit (TaKaRa) on an ABI Prism 7500 real-time PCR system (Applied Biosystems) following the manufacturer’s protocol. Quantitative mRNA expression was measured with ABI Prism 7500 Software v2.0.6 and calculated based on the CT values normalized to human β-actin expression. The primer sequences are shown in Table S1.

### Immunofluorescent staining

Serial paraffin sections (4 μm) from human CSCC tissues were analysed by immunofluorescence with the Opal 4-Color Kit (PerkinElmer) according to the manufacturer’s protocol. Briefly, sections were microwaved in antigen retrieval buffer for 15 min at 90 °C after deparaffinization; then, they were washed and blocked for 10 min at 26 °C and incubated with the first primary antibody. Horseradish peroxidase-conjugated secondary antibody was dropped onto slides for incubation for 10 min at 26 °C. Subsequently, tyramide signal amplification (TSA) buffer (Opal 650) was used to amplify the signal on the slides. After eliminating the primary and secondary antibodies by microwaving, the above procedures were repeated with the second primary antibody and TSA buffer (Opal 570). The above procedures were repeated again with the third primary antibody and TSA buffer (Opal 520). The primary antibodies include anti-CAIX (#ab184006; applied at 1:1000; Abcam), anti-LV endothelial hyaluronan receptor-1 (LYVE-1) (#ab33682; applied at 1:1000; Abcam), anti-CD163 (#ab156769; applied at 1:1000; Abcam; a TAM marker for human [20]), CD206 (#ab64693; applied at 1:1000; Abcam; a TAM marker for mouse [21]), anti-Sp1 (#sc-420; applied at 1:100; Santa Cruz Biotechnology) and anti-CCL1 (#DF9910; applied at 1:500; Affinity). The sections were mounted in neutral gum and visualized by a fluorescence microscope (Olympus).

### Transwell migration assay

For the M2-polarized THP-1 macrophages, THP-1 cells were treated with 5 nM phorbol 12-myristate 13-acetate (PMA)(Sigma-Aldrich, Shanghai, P.R. China) for 6 h, followed by culture with PMA supplemented with 20 ng/ml interleukin (IL)-4 and 20 ng/ml IL-13 for another 18 h [22]. Transwell assays to assess cell migration potential were performed on 24-well plates with inserts (8-μm pore size; Millipore, Billerica, MA, USA), as previously described [23]. Briefly, 5 × 10^5^ tumour cells (SiHa) or M2-polarized THP-1 macrophages were cultured in the upper chamber. Different CM were placed in the lower chamber and allowed to migrate for 24 h before fixation for Crystal Purple staining. For CM collection, HDLECs were cultured in different macrophage supernatants for 48 h, and after washing in fresh medium, cells were incubated for another 48 h in serum-free medium at 37 °C. CM was then harvested, centrifuged, and used for the transwell migration assay. Cytokine concentrations were set according to the migration effect of activated LEC CM. The migration effect of TAM-H-treated LECs on tumour cells and M2 macrophages was almost equivalent to that observed with 50 ng/ml IL-10-treated LECs and 50 ng/ml CCL1. Cells were counted in five random fields of view. Data represent the mean ± standard deviation, and experiments were at least in triplicate.

### Immunohistochemistry (IHC)

Tissue sections were subjected to IHC analysis as previously described [24]. Briefly, consecutive 4-μm paraffin sections were deparaffinized and rehydrated. Endogenous peroxidase activity was blocked by incubation for 30 min with 3% H_2_O_2_ in methanol at 26 °C. Antigen retrieval was performed using a microwave treatment for 15 min in citrate buffer (pH 6.0). The sections were then blocked with 10% goat serum at 37 °C for 1 h and incubated with primary antibodies against the primary antibody in a humidified chamber overnight at 4 °C. Next, the sections were incubated with the secondary antibody and processed with the universal SP histostain™-plus kit (ZYMED, Carlsbad, CA, USA). Finally, the slides were counterstained with hematoxylin. The primary antibodies include CAIX (#ab184006; applied at 1:500; Abcam), LYVE-1 (#ab33682; applied at 1:100; Abcam), CD163 (#ab156769; applied at 1:1000; Abcam), CK7 (#17,513–1-AP; applied at 1:1000; Proteintech), IL-10 (#DF6894; applied at 1:1000; Affinity), CCL1 (#DF9910; applied at 1:1000; Affinity) and Sp1 (#sc-420; applied at 1:100; Santa Cruz Biotechnology). The secondary antibodies were horseradish peroxidase-conjugated anti-rabbit and mouse immunoglobulin-G antibody (both applied at 1:5000, Abcam).

### Evaluation of IHC results

The counting was done using the H score algorithm [25]. For H score assessment, fields were at ×400 magnification, and the staining intensity in the malignant cell was scored as 0, 1, 2, or 3, corresponding to the presence of negative, weak, intermediate, and strong brown staining, respectively. The total number of cells in each field and the number of cells stained at each intensity were counted. H score was calculated as follows: (% of cells stained at intensity 1 × 1) + (% of cells stained at intensity 2 × 2) + (% of cells stained at intensity 3 × 3). An H score between 0 and 300 was obtained, where 300 was equal to 100% of tumour cells stained strongly (3+). The median H score values were selected for distinction between the groups of low and high CAIX, IL-10, Sp1 or CCL1 expression. The densities of TAMs and LVs were counted as previously described [19, 24].

### Enzyme-linked immunosorbent (ELISA) assay

CCL1 and IL-10 levels in the CM of HDLECs were measured using ELISA kits (#EHCCL1, Thermo Scientific; CSB-E04594m, CUSABIO) according to the manufacturer’s instructions. Briefly, a 96-well microplate was precoated with the primary antibody. First, 100 µl of each standard or sample was added to the appropriate wells and incubated for 2 h at 26℃ with gentle shaking. After discarding the solution and washing four times, 100 µl of detection antibody was added to each well and incubated for 1 h. After washing away the unbound biotinylated antibody, 100 µl of horseradish peroxidase (HRP)-conjugated streptavidin was added to the wells and incubated for 30 min, and 100 µl of tetramethylbenzidine (TMB) one-step substrate reagent was added after five washes. Subsequently, 50 µl of stop solution was added to each well, and the plate was immediately read at 450 nm.

### Dual-luciferase assays

The expression of Sp1 targeted gene was measured using a dual-luciferase reporter assay in HDLECs and 293T cells according to the manufacturer’s instructions. Briefly, co-transfection of Sp1 or empty plasmid with CCL1 promoter into the cells was accomplished using Lipofectamine™ 2000 (Invitrogen, USA). Luciferase activity was measured 48 h after transfection by the Dual-Luciferase Reporter Assay System. Each assay was repeated in 3 independent experiments.

### Data source

The gene expression dataset from CSCC patients was obtained from the University of California, Santa Cruz Xena browser (UCSC Xena: http://xena.ucsc.edu/, accessed January 7, 2019). The corresponding clinical information of CSCC patients was downloaded from The Cancer Genome Atlas (TCGA, https://portal.gdc. cancer.gov/, accessed January 7, 2019). The datasets included in the current study were downloaded from public databases; therefore, there was no need for the study to be approved by an additional ethics committee.

### Statistics

SPSS (version 20.0) software was used for statistical analysis. The results are expressed as the mean value ± SEM and were interpreted by the *t*-test. Frequency tables were analysed using the Chi-squared test, and the Pearson’s correlation coefficient was used to assess the significance of the correlations between categorical variables. Univariate survival analysis of IL-10, Sp1 and CCL1 were analysed using the log-rank test. Differences were considered to be statistically significant when *P* < 0.05.

### Study approval

All experimental protocols were approved by The IACUC (Institutional Animal Care and Use Committee) of Guangzhou Medical University, and the study was carried out in strict accordance with the Animal Research: Reporting In Vivo Experiments (ARRIVE) guidelines [26]. For human samples, all CSCC samples were acquired from the First Affiliated Hospital of Guangzhou Medical University. The procedures related to human subjects were approved by Ethic Committee of Guangzhou Medical University.

## Results

### LVEM represents a novel lymphatic pattern correlated with lymphatic metastasis of CSCC

**Fig. 1:**
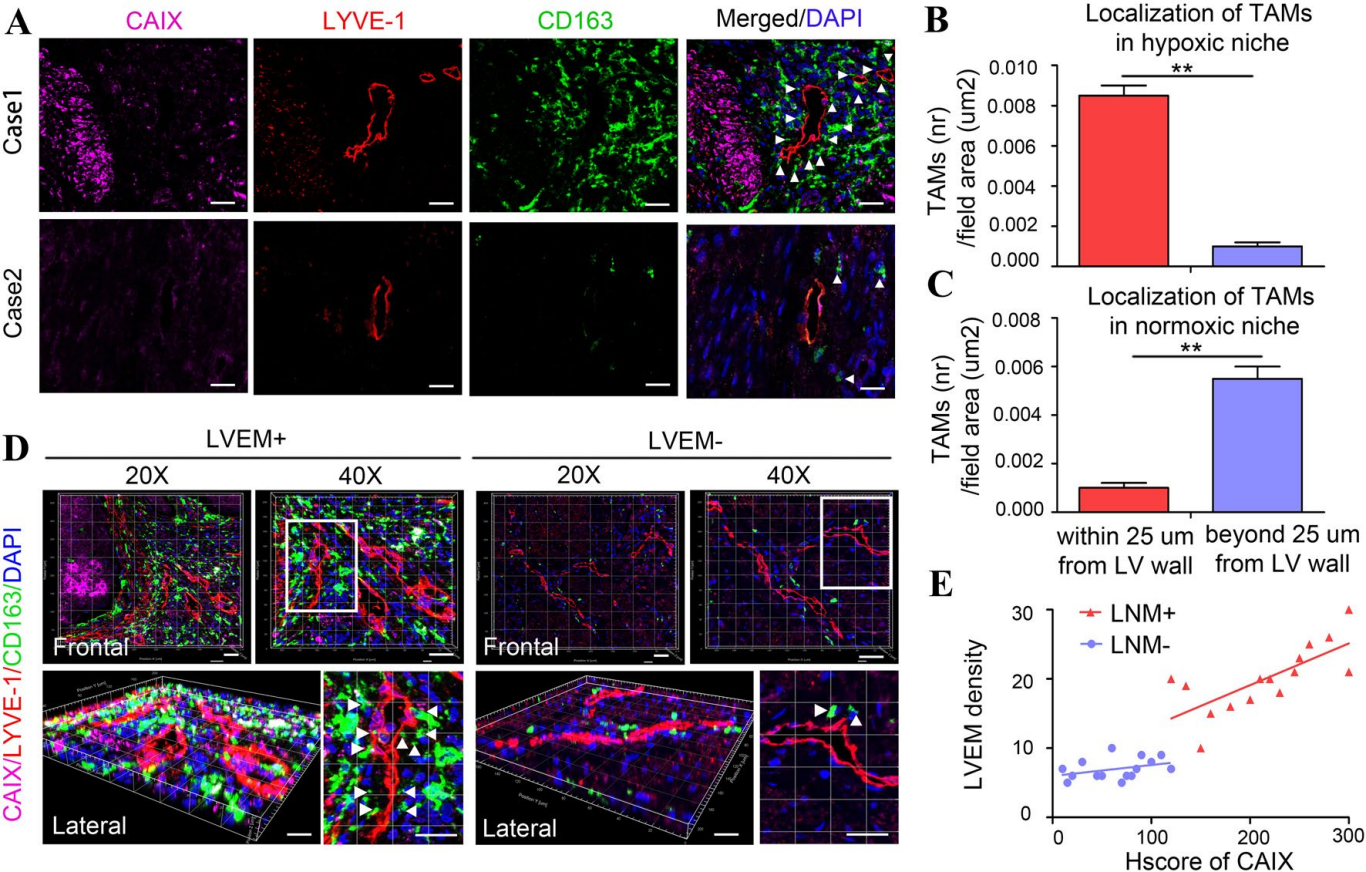
LVEM represents a novel lymphatic subtype correlated with lymphatic metastasis of CSCC. **a** Representative images of CAIX (purple), LYVE-1^+^ lymphatic vessel (red), CD163^+^ TAMs (green) and DAPI (blue) fluorescence staining in CSCC tissues. Images are shown at × 400 magnification (Scale bar, 50 μm). **b**–**c** Statistical analysis of the localization of CD163^+^ TAMs to LYVE-1^+^ lymphatic vessels (within or beyond a distance of 25 μm from lymphatic vessel) in hypoxic (**b**) and normoxic (**c**) regions. The number of cells was normalized per counting area (μm^2^). **d** Three-dimensional reconstruction of the lymphatic architecture. Representative images indicate two distinct LVs in CSCC tissues (outline in white box and amplify in the lower right corner). Images are shown at ×200 and ×400 magnification respectively (Scale bar, 50 μm). **e** The correlation between CAIX expression and LVEM density was statistically analysed by Pearson’s coefficient test (red line, *r* = 0.5240, *P* = 0.0023; blue line, *r* = 0.1335, *P* = 0.1804)

Using immunofluorescent staining for the lymphatic marker LYVE-1 in human CSCC tissues, we observed two distinct lymphatic patterns: LVs encapsulated by considerable CD163^+^ TAMs in carbonic anhydrase IX (CAIX; an established cellular biomarker of hypoxia)—positive hypoxic regions and LVs with few CD163^+^ TAMs in CAIX-negative normoxic regions (Fig. 1a). Histologic assessment revealed that TAMs in hypoxic niche were more proximal to the LVs than TAMs in normoxic niche (Fig. 1b–c). Moreover, there was a higher LV density surrounding hypoxic TAMs than normoxic TAMs (Fig. S1). And confocal imaging of thick sections from CSCC tissues confirmed that hypoxic TAMs localized in the proximity of or adhered to LVs (Fig. 1d). We designated cases displaying LVEM in most of the sections as LVEM^high^ and those with few or no LVEM as LVEM^low^. Three-dimensional reconstruction confirmed that LVEM developed into an interconnected network in the hypoxic TME encapsulated by TAMs, whereas inactivated LVs showed discrete and disorganized patterns in normoxic regions. Additionally, LVEM^high^ patients showed significantly higher rates of microemboli as compared with LVEM^low^ cases (Fig. 1d). Clinical relevance analysed by Pearson’s coefficient test indicated that higher CAIX level strongly correlated with increments of LVEM density in CSCC^LNM^ tissues (*r* = 0.5240, *P* = 0.0023) (Fig. 1e, red line), but no correlation in CSCC^non-LNM^ tissues (*r* = 0.1335, *P* = 0.1804) (Fig. 1e, blue line). These results suggested that the LVEM was prevalent in hypoxic regions of CSCC^LNM^ tissues.

To evaluate the clinical value of the LVEM, we examined relationships among CAIX level, LV density, LVEM density, and clinicopathologic features. We found that upregulated CAIX level, enhanced LV intensity, and increased LVEM intensity were strongly correlated with LNM and advanced FIGO stage of CSCC (*P* < 0.05) (Table 1). Additionally, CAIX level and LVEM were significantly associated with lymphovascular space invasion (LVSI) (*P* < 0.05) (Table 1). These data implied that hypoxia is closely related to lymphatic metastasis and poor prognosis in CSCC patients, with this possibly mediated by LVEM-associated LVSI.

### Hypoxic TAMs promote lymphangiogenesis and LVEM formation

**Fig. 2:**
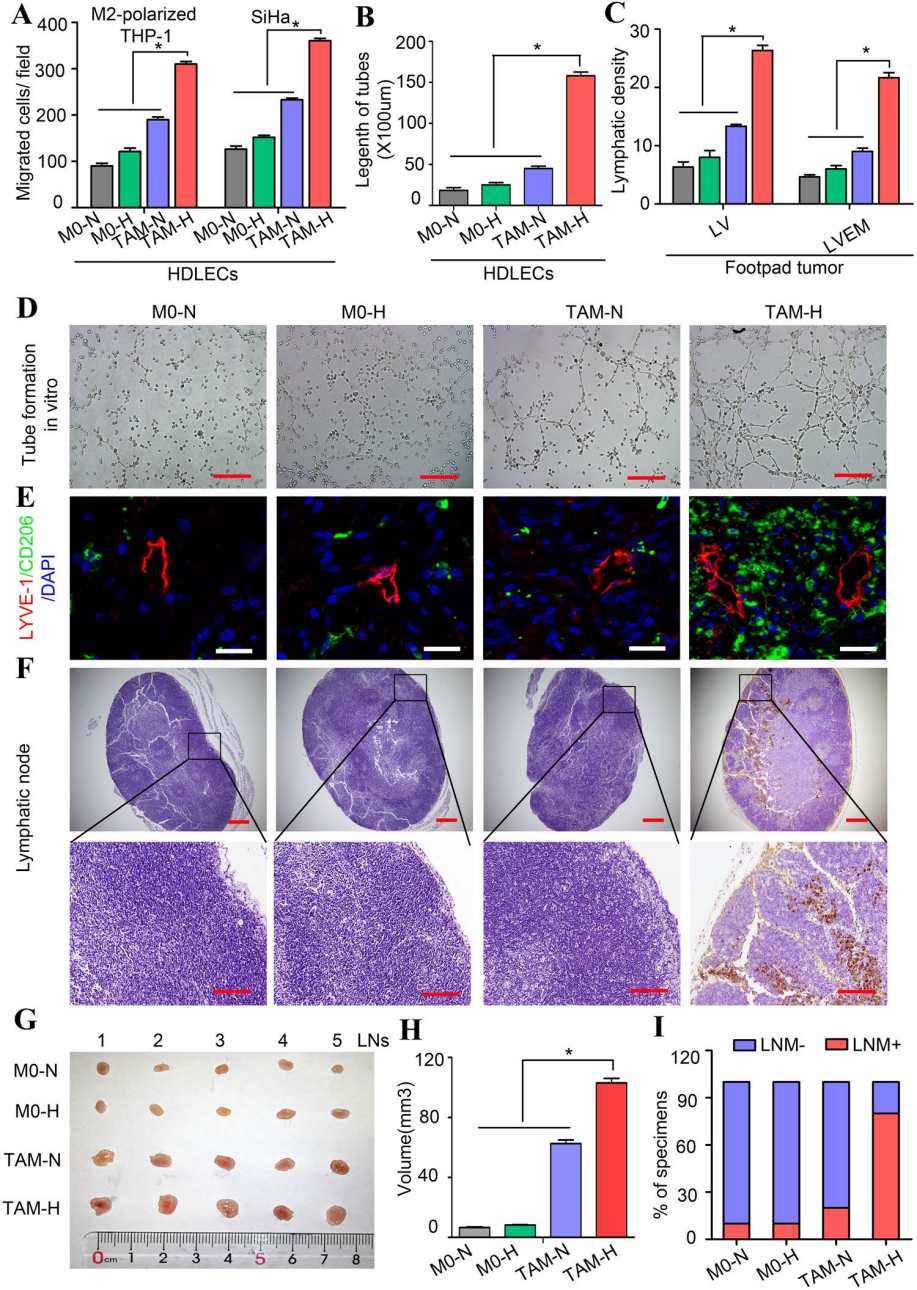
Hypoxic TAMs promote lymphangiogenesis and LVEM formation. **a** The function of CM from different macrophage-treated HDLECs on M2-polarized THP-1 macrophages and tumour cells (SiHa) was detected by transwell array in vitro. **b** Statistical analysis showing the length of tube formation in vitro. Average length of tubes per field were calculated. **c** Statistical analysis showing the expression of LV and LVEM in mouse footpad tumour. **d** Representative micrographs showing tube formation assay in vitro of HDLECs pretreated with different macrophage CM for 48 h. Scale bar, 50 μm. **e**–**i** Popliteal lymphatic metastasis model was established in female C57BL/6 mice by inoculating the footpad with TC-1 cells (5 × 10^6^). When footpad tumour size reached 50 mm^3^, macrophage supernatants of different treatment conditions (10 μl) were then injected into the centre of the tumours (*n* = 5/group, repeated twice) for 2 weeks daily. After 2 weeks of induction, primary tumours reached a comparable size of ~ 150 mm^3^, and then footpad tumours and popliteal LNs were collected for study. **e** Representative images of LYVE-1^+^ lymphatic vessel (red), CD206^+^ TAMs (green) and DAPI (blue) fluorescence staining in footpad tumour. Images are shown at × 400 magnification (Scale bar, 50 μm). **f** IHC Staining of CK7 in popliteal LNs. Representative micrographs are shown (Scale bar, 100 μm). Metastasis-positive LNs were identified by staining for epithelial marker CK7. **g** Photos of mouse popliteal LNs in different macrophage CM-primed tumour (*n* = 5/group). **h** Statistical analysis showing the volume (mm^3^) of the LNs. **i** The ratio of metastasis-positive to total dissected popliteal LNs from mice treated with different macrophage supernatants. Error bars represent the mean ± SD of three independent experiments. *P < 0.05. N: Normoxia; H: Hypoxia

Our previous study reported that TAMs in the hypoxic TME exert immunosuppressive effects with a tumour-supportive role [12]. To investigate how the LVEM was induced, we cultured HDLECs with different macrophage CM (M0-N, M0-H, TAM-N, and TAM-H) for 48 h. We found that CM from TAM-H-treated HDLECs recruited more M2 macrophages and SiHa cells relative to other groups (*P* < 0.05) (Fig. 2a). To investigate the role of TAMs in lymphangiogenesis, we analysed tube formation by HDLECs incubated with different macrophage CM for 48 h, finding that TAM-H dramatically increased the number and length of tubes formed relative to that observed in other groups (*P* < 0.05) (Fig. 2b and d). We then examined the pro-metastatic role of TAM-H by employing a mouse popliteal lymphatic metastasis model, which simulates the directional drainage and LNM of CSCC. We found that the density of LVs was higher in footpad tumours of the TAM-H group as compared with that in other groups (*P* < 0.05) (Fig. 2c and e). Moreover, considerable CD206^+^ TAMs infiltrated to the stroma surrounding the LVs in the TAM-H-CM-primed tumours, whereas this was not observed in the other groups. Statistical analysis revealed that LVEM density was higher in TAM-H-CM-primed tumours than other groups (*P* < 0.05) (Fig. 2c and e). We then evaluated the effect of TAM-H CM on lymphatic metastasis after control tumours reached the same size as experimental tumours. The results showed that TAM-H CM promoted tumour cells to metastasis to LNs according to immunochemistry of CK7 (an epithelial marker) (*P* < 0.05) (Fig. 2f and i). Additionally, LN volume was larger in TAM-H-CM-primed tumours relative to that in other groups (*P* < 0.05) (Fig. 2g and h). These results suggested that the LVEM is remodelled by a subset of hypoxic TAMs adjacent to LVs.

To determine whether macrophage infiltration is involved in LVEM formation and LNM, TAMs were depleted using CLs following subcutaneous injection of TC-1 cells in the mouse footpad. CL treatment significantly decreased CD206^+^ TAMs in the primary tumour relative to levels observed after treatment with control liposomes (Fig. S2A and D). Moreover, in vivo assays showed that CL treatment dramatically decreased LV density, the occurrence of LVEM, and the number of metastatic LNs (Fig. S2B–D). Furthermore, LN volumes were smaller in the CL treatment group than the control group (*P* < 0.05) (Fig. S2E and F). These results suggested that TAMs facilitated LVEM formation and metastatic progression.

### IL-10 derived from hypoxic TAMs is required to maintain the LVEM

**Fig. 3:**
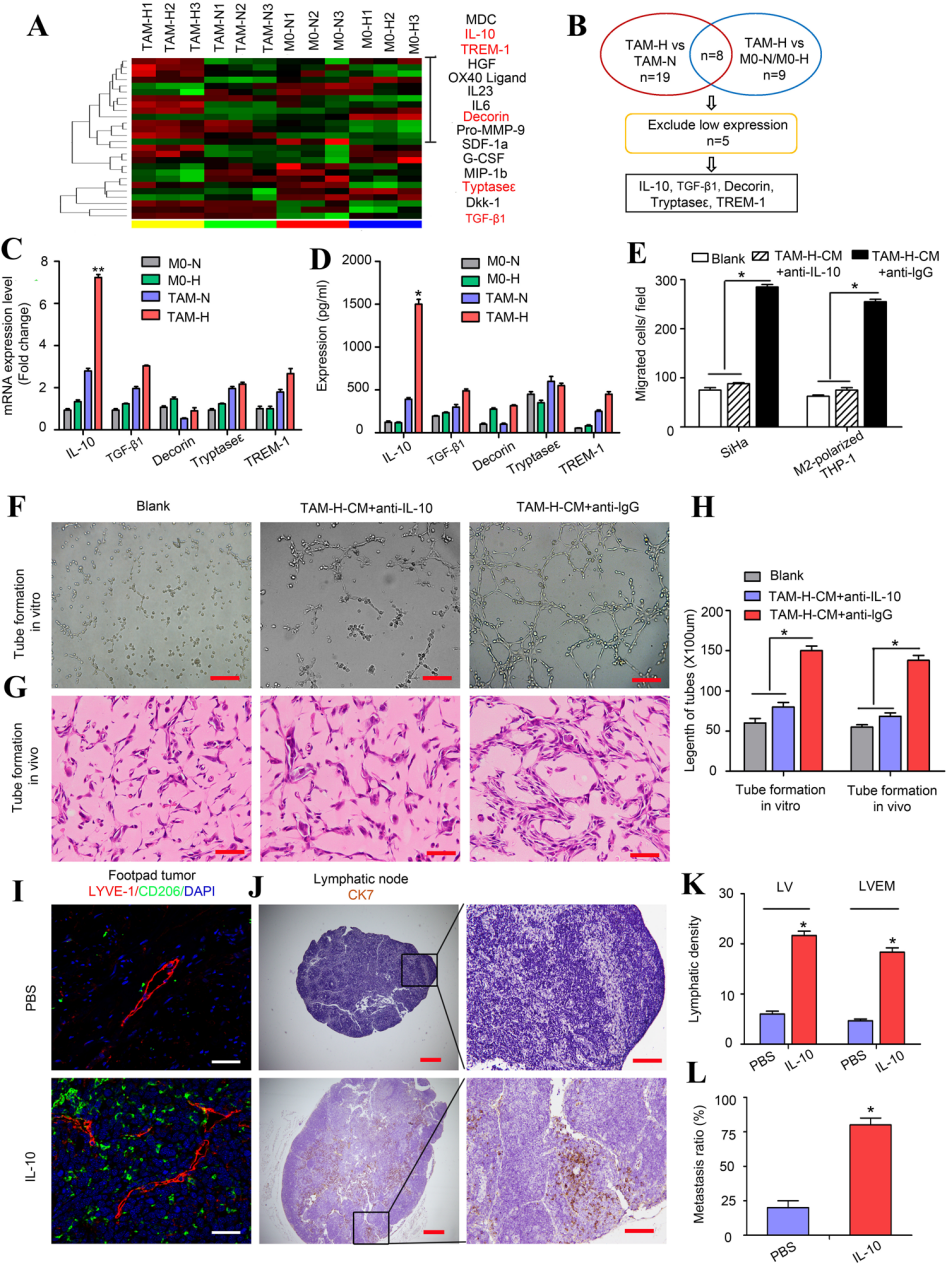
IL-10 derived from hypoxic TAMs is required to maintain LVEM. **a** The different cytokines expression profiles among M0-N, M0-H, TAM-N and TAM-H were analysed by cytokine array (RayBio GSM-CAA-4000). **b** Screening and analysis of the differentially expressed cytokines. **c** The expressions of the five significant cytokines were analysed by qRT-PCR. **d** The secretions of the five significant cytokines were analysed by ELISA. **e** The migration effects of hypoxic TAMs-treated HDLECs on tumour cells (SiHa) and M2-polarized THP-1 macrophages were analysed by transwell assay in vitro. “Blank” represents the medium group. **f** Representative micrographs showing the tube formation in vitro (Scale bar, 50 μm). **g** Representative images showing the tube formation in vivo (Scale bar, 100 μm). **h** Statistical analysis showing the length of tube formation. Average length of tubes per field were calculated. **i**–**l** Popliteal lymphatic metastasis model was established in female C57BL/6 mice by inoculating the footpad with TC-1 cells (5 × 10^6^). When footpad tumour size reached 50 mm^3^, IL-10 (50 ng/ml) or PBS was then injected into the centre of the tumours (*n* = 5/group, repeated twice) for 2 weeks daily. After 2 weeks of induction, primary tumours reached a comparable size of ~ 150 mm^3^, and then footpad tumours and popliteal LNs were collected for study. **i** Representative images of LYVE-1^+^ lymphatic vessel (red), CD206^+^ TAMs (green) and DAPI (blue) fluorescence staining in footpad tumour. **j** Metastasis-positive LNs were identified by IHC staining for epithelial marker CK7. **k** Statistical analysis showing the expression of peritumoural LV and LVEM in footpad tumour. **l** Statistical analysis showing the ratio of LNM. Error bars represent the mean ± SD of three independent experiments. **P < 0.01

TAMs in the hypoxic milieu displayed a more deadly and complicated cytokine-expression unit than in the normoxic tumour area in order to modulate TME suitability for cancer progression [11]. To elucidate the factors involved in hypoxic TAM-mediated LVEM formation, we performed analyses using a cytokine array (Fig. 3a). A search for differentially expressed cytokines identified five, including IL-10, transforming growth factor β1 (TGF-β1), decorin, tryptase ε, and triggering receptor expressed on myeloid cells-1 (TREM-1), that were significantly upregulated in TAM-H relative to TAM-N and the control macrophages (Fig. 3b). Among these, IL-10 was confirmed as displaying the most significant differential expression according to qRT-PCR and ELISA assay (**P < 0.01 and *P < 0.05, respectively) (Fig. 3c and d). To examine IL-10 expression in CSCC tissues, 75 primary tumours were subjected to triple-label immunofluorescence for CAIX, CD163, and IL-10 (Fig. S3A). The results showed co-localization of IL-10 and CD163 according to immunostaining in CAIX-positive hypoxic tissues, confirming that IL-10-positive cells were subsets of hypoxic TAMs, whereas only scattered staining was observed in neoplastic cells or normoxic TAMs. Moreover, we identified a significant correlation between CD163 and IL-10 expression in hypoxic tissues, whereas this was not observed in normoxic tissues (*r* = 0.5178, *P* < 0.0001; and *r* = 0.1253, *P* = 0.0550, respectively) (Fig. S3B), and CD163^+^ TAMs and IL-10 expression were more abundant in hypoxic regions of CSCC tissues than in normoxic regions (*P* < 0.05) (Fig. S3C).

We observed that the migration effects of TAM-H-treated HDLECs on tumour cells (SiHa) and M2-polarized THP-1 macrophages were significantly decreased by adding anti-IL-10 neutralization antibody to the CM (*P* < 0.05) (Fig. 3e). Additionally, lymphangiogenesis and LVEM density were significantly decreased in TAM-H-CM + anti-IL-10-pretreated HDLECs relative to those pretreated with PBS or anti-lgG according to tube formation assays in vitro and in vivo (*P* < 0.05) (Fig. 3f–h). Assessment of the effect of IL-10 on lymphatic metastasis in vivo using a popliteal lymphatic metastasis model revealed that IL-10 significantly promoted lymphangiogenesis and LVEM formation (*P* < 0.05) (Fig. 3i and k), with a higher ratio of metastasis-positive popliteal LNs found in IL-10-pretreated groups relative to PBS-pretreated groups (*P* < 0.05) (Fig. 3j and l). These data suggested IL-10 as a critical mediator of cross-talk between hypoxic TAMs and LECs.

### CCL1 mediates TAM migration towards LECs during LVEM formation

**Fig. 4:**
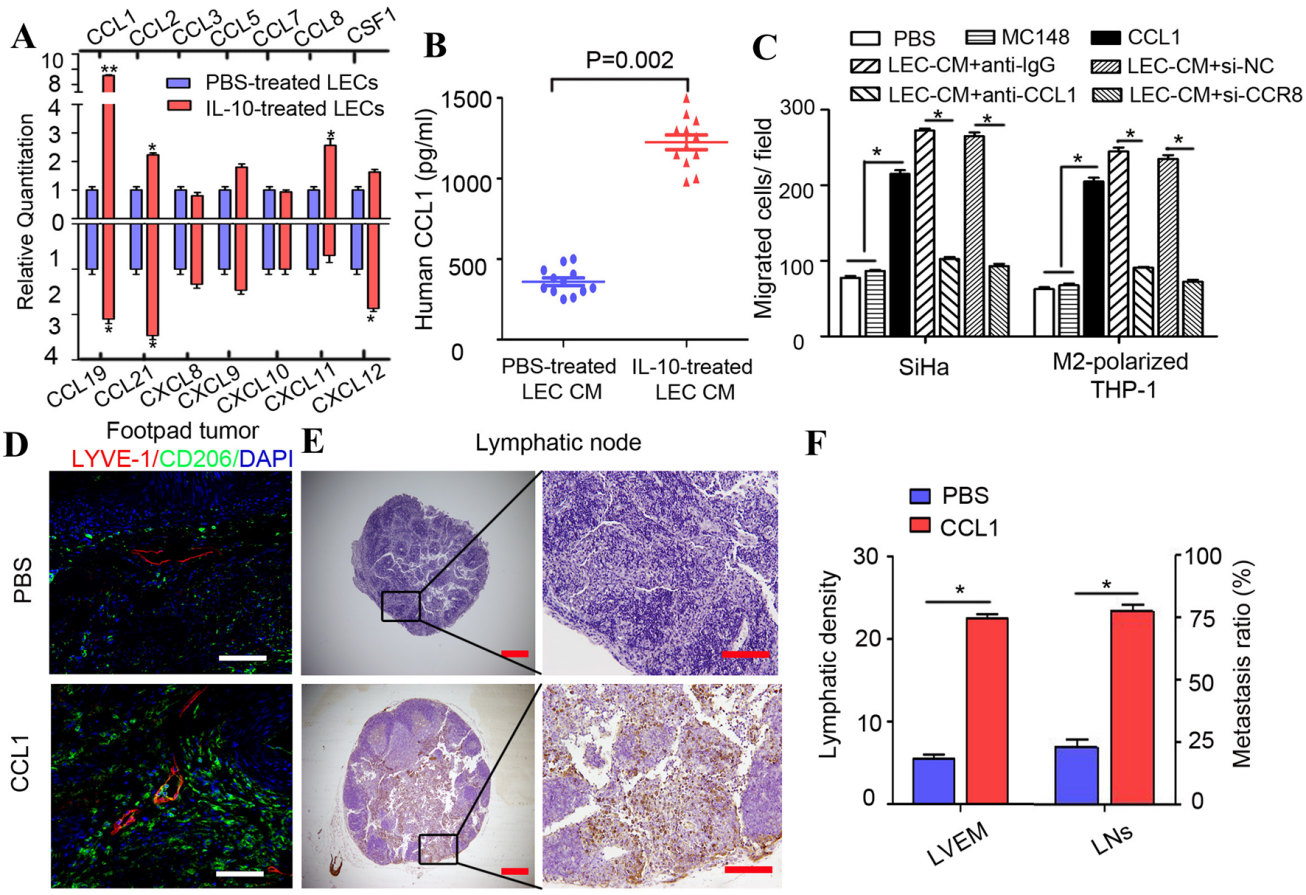
CCL1 mediates the migration of TAMs towards LECs in the formation of LVEM. **a** Multiple related chemokines were screened in IL-10-activated LECs by qRT-PCR. **b** Levels of CCL1 secreted by IL-10-activated LECs were measured by ELISA. **c** The migration effects of CCL1 on tumour cells (SiHa) and M2-polarized THP-1 macrophages were significantly decreased by blockade of CCR8 on receptor cells (siRNA or CCR8 antagonist MC148) or adding anti-CCL1 neutralization antibody in IL-10-activated LEC CM. **d**–**f** Popliteal lymphatic metastasis model was established in female C57BL/6 mice by inoculating the footpad with TC-1 cells (5 × 10^6^). When footpad tumour size reached 50 mm^3^, CCL1 (50 ng/ml) or PBS was then injected into the centre of the tumours (*n* = 5/group, repeated twice) for 2 weeks daily. After 2 weeks of induction, primary tumours reached a comparable size of ~ 150 mm^3^, and then popliteal LNs were collected for study. **d** Representative images of LYVE-1^+^ lymphatic vessel (red), CD206^+^ TAMs (green) and DAPI (blue) fluorescence staining in footpad tumour. **e** IHC Staining of CK7 in popliteal LNs from mice treated with CCL1 (Scale bar, 100 μm). **f** Statistical analysis showing the ratio of LNM. Error bars represent the mean ± SD of three independent experiments. **P < 0.01. *P < 0.05

Chemokines are necessary for macrophage migration and tumour cell dissemination [15]. To identify the chemokines secreted by IL-10-activated LECs and that promoted LVEM formation, we evaluated serial chemokine expression by qRT-PCR and ELISA assays. Among the upregulated chemokines (CCL1, CCL2, CCL8, CCL19, CCL21, and CXCL12), CCL1 levels showed the highest elevation according to qRT-PCR (*P* < 0.05) (Fig. 4a), and ELISA assay consistently identified increased CCL1 levels in the CM of IL-10-activated HDLECs (Fig. 4b). MC148 is the competitive CCL1 antagonist that binds C–C motif chemokine receptor 8 (CCR8) with the same affinity as its endogenous ligand CCL1. Pre-incubation of SiHa cells and M2-polarized THP-1 macrophages with MC148, blockade of CCR8 with siRNA, or adding an anti-CCL1 neutralization antibody to IL-10-activated LEC CM significantly impaired cell migration, indicating that the CCL1–CCR8 axis mediates most of the chemotactic response to LEC CM (*P* < 0.05) (Fig. 4c). We then examined whether CCL1 induces lymphatic metastasis in vivo using a popliteal lymphatic metastasis model. Treatment with CCL1 significantly increased the LVEM and the ability of TC-1 cells to metastasize to LNs according to CK7 immunochemistry (Fig. 4d–f). These data identified CCL1 as a potent tumour chemotactic factor produced by LECs and critically involved in LVEM-induced lymphatic metastasis.

### Sp1^high^ LECs are fundamental to LVEM formation and lymphatic metastasis

**Fig. 5:**
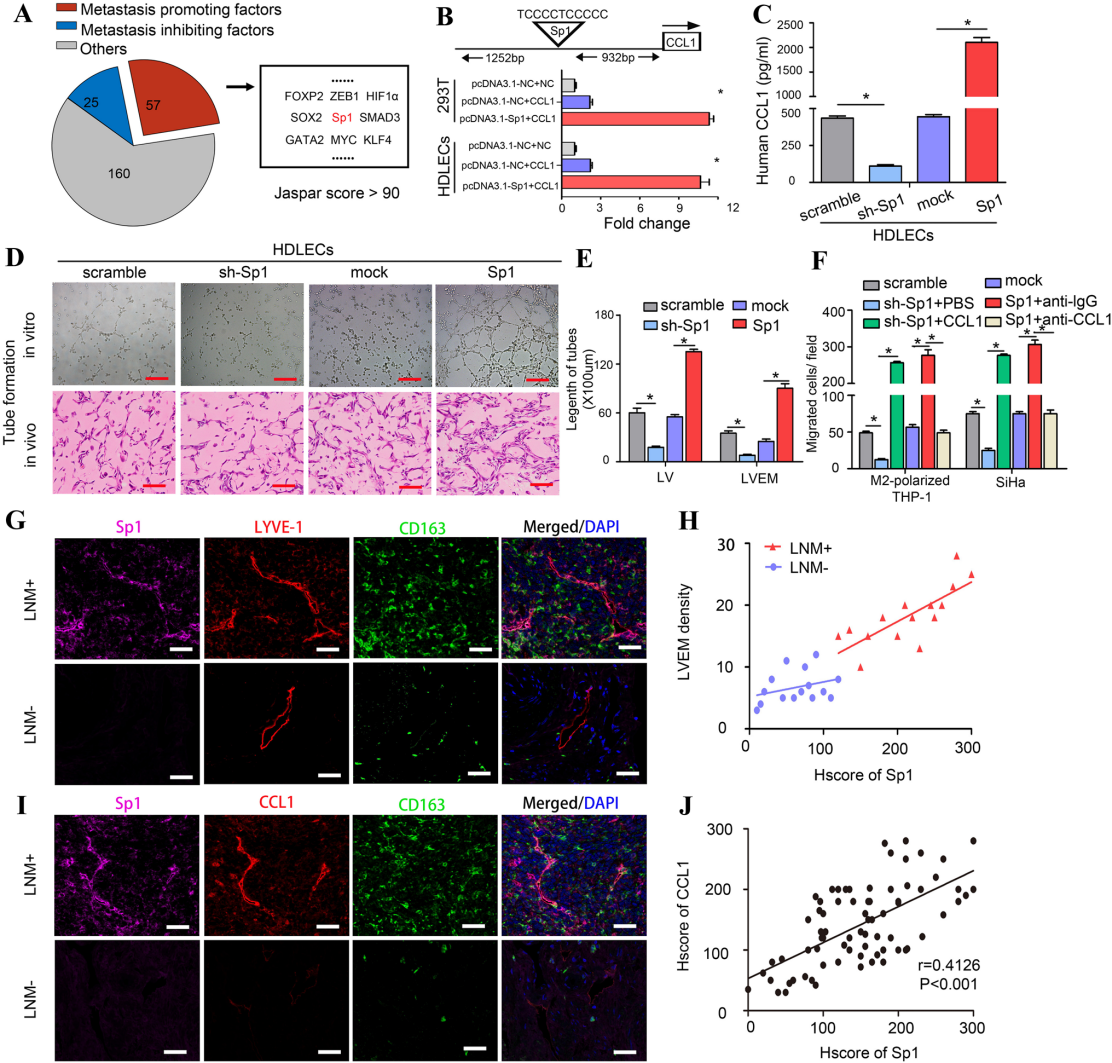
Sp1^high^ LECs are fundamental to LVEM formation and lymphatic metastasis. **a** Bioinformatic prediction and screening of potential transcription factors of CCL1. **b** RNA sequence of the predicted Sp1-binding site to CCL1 was TCCCCTCCCCC. A dual-luciferase reporter assay system was performed to demonstrate the direct binding of Sp1 to the CCL1 promoter region. Transient co-transfection of pcDNA3.1-Sp1 construct with CCL1 promoter into both 293T cells and HDLECs resulted in a significant increase in firefly luciferase activity relative to co-transfection of pcDNA3.1-NC construct with CCL1 promoter or the negative control. **c** EILSA analysis showed that CCL1 upregulation was detected in Sp1-transduced HDLEC CM, and CCL1 was decreased in Sp1-silenced HDLEC CM. **d** Representative micrographs of the tube formation assay in vitro (upper panel) and in vivo (lower panel) of HDLECs with stable overexpression or silencing of Sp1 (Scale bar, 100 μm). **e** Statistical analysis showing the length of tube formation. Average length of tubes per field were calculated. **f** The migration effects of M2-polarized THP-1 macrophages and tumour cells (SiHa) towards CM of HDLECs with stable overexpression or silencing of Sp1 were detected by transwell array in vitro. **g** Immunofluorescence staining was applied to analyse Sp1 (purple), LYVE-1 (red), CD163 (green) and DAPI (blue) expression in CSCC tissues (Scale bar, 100 μm). **h** The correlation between Sp1 expression and LVEM density was statistically analysed by Pearson’s coefficient test (red line, *r* = 0.5846, *P* = 0.0009; blue line, *r* = 0.1018, *P* = 0.2465). **i** Immunofluorescence staining was applied to analyse Sp1 (purple), CCL1 (red), CD163 (green) and DAPI (blue) expression in CSCC tissues (Scale bar, 50 μm). **j** The correlation between Sp1 and CCL1 expression was statistically analysed by Pearson’s coefficient test (*r* = 0.4126, *P* < 0.001). Error bars represent the mean ± SD of three independent experiments. *, *P* < 0.05

The malignant characteristics of LVEM drove us to further explore its specific phenotype. To investigate the transcription factors (TFs) targeting the CCL1 promoter, we scanned the 2 000-bp sequence for TF-binding site motifs using the JASPAR database (http://jaspar.genereg.net/). A total of 242 genes with a Jaspar score > 90 were selected, and a literature search confirmed 57 as potential metastasis-promoting genes (Fig. 5a), including Sp1, a master regulator of cancer metastasis [27]. Our previous studies reported that elevated expression of Sp1 is closely correlated with LNM [28], which could have significant implications for its potential utility as a metastasis-associated biomarker of CSCC. The RNA sequence of the predicted Sp1-binding site in the CCL1 promoter is TCCCCTCCCCC (Fig. 5b), which suggested Sp1 as a putative upstream TF for CCL1. We then performed a dual-luciferase reporter assay to demonstrate the direct binding of Sp1 to the CCL1 promoter region. Transient co-transfection of pcDNA3.1-Sp1 construct with CCL1 promoter into both 293T cells and HDLECs resulted in a significant increase in firefly luciferase activity relative to that observed in controls (*P* < 0.05) (Fig. 5b). Additionally, ELISA revealed CCL1 upregulation in Sp1-transduced HDLEC CM, whereas CCL1 levels were decreased in sh-Sp1-transduced HDLEC CM (*P* < 0.05) (Fig. 5c). These results suggested that Sp1 regulates CCL1 expression at the transcriptional level.

We then established HDLECs stably overexpressing or silencing Sp1 in order to investigate whether Sp1 is essential for LVEM formation (Fig. S4). The results showed that lymphangiogenesis and LVEM significantly increased in Sp1-overexpressing HDLECs relative to the Sp1-silenced group according to tube formation assays in vitro and in vivo (Fig. 5d and e). Silencing Sp1 in HDLECs resulted in decreased recruitment of M2-polarized THP-1 macrophages and tumour cells (SiHa), whereas Sp1 overexpression increased cell migration. Additionally, CCL1 increased sh-Sp1-reduced cell migration, whereas anti-CCL1 neutralization antibody decreased Sp1-enhanced cell migration (*P* < 0.05) (Fig. 5f). Consistent with the result of transwell assay in vitro, impaired TAM adhesion to LECs resulted in a decreased metastatic ratio in sh-Sp1 popliteal LNM model or Sp1 overexpression model adding with anti-CCL1 neutralization antibody, whereas the model displaying Sp1 overexpression and Sp1-silenced adding with CCL1 showed significantly induced LNM (*P* < 0.05) (Fig. S5F). Immunofluorescence staining to analyse Sp1, CCL1, LYVE-1, and CD163 expression on tissues derived from CSCC patients indicated that compared with LVs in CSCC^non-LNM^ tissues, Sp1 level in CSCC^LNM^-associated LVs increased significantly, with the Sp1 signal mainly detected in LVs of LVEM^high^ tissues (Fig. 5g). Furthermore, we found a significant correlation between Sp1 expression and LVEM density in CSCC^LNM^ tissues (*r* = 0.5846, *P* = 0.0009) (Fig. 5h, red line), whereas no correlation was observed in CSCC^non-LNM^ tissues (*r* = 0.1018, *P* = 0.2465) (Fig. 5h, blue line). Additionally, Sp1^+^ CCL1^+^ LVs were surrounded by CD163^+^ TAMs in CSCC^LNM^ tissues, whereas it is undetectable in CSCC^non-LNM^ tissues (Fig. 5i). Analysis of clinical relevance according to Pearson’s coefficient test indicated a significant correlation between Sp1 and CCL1 expression (*r* = 0.4126, *P* < 0.001) (Fig. 5j).

We then elucidated the signalling pathway involved in LVEM formation by western blot and immunofluorescence. The results revealed that incubation of HDLECs with IL-10 led to increased expression of Sp1 via activation of p-STAT3 signalling (Fig. S5A, B, and G). Following inhibition of STAT3 signalling using 5,15-diphenyl-porphine or the anti-IL-10R neutralization antibody, we observed obvious decreases in Sp1 expression on LECs and LEC attraction of M2-polarized THP-1 macrophages and tumour cells (SiHa) (Fig. S5C–E). Additionally, Sp1 downregulation in HDLECs decreased IL-10-enhanced cell migration (*P* < 0.01) (Fig. S5E). These findings indicated that Sp1^high^ LECs are essential for LVEM formation, and the double staining of Sp1 and CCL1 might serve as specific markers for metastasis-associated LVs.

### Potential clinical relevance of the LVEM in CSCC patients

**Fig. 6:**
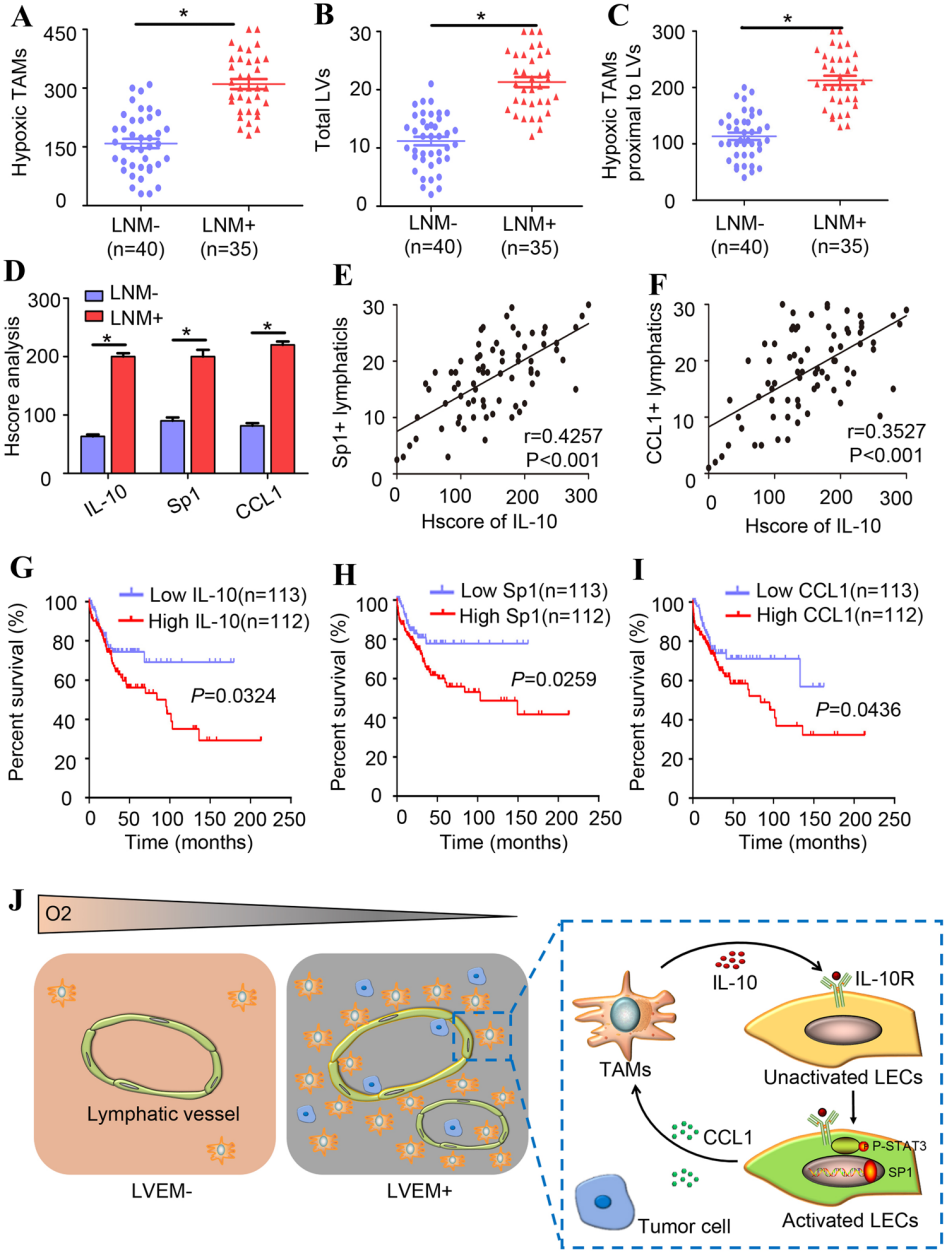
Potential clinical relevance of LVEM in cervical cancer patients. The number of hypoxic TAMs (**a**), peritumoural LV density (**b**) and hypoxic TAMs per tumour LV (**c**) were analysed in CSCCLNM tissues (n = 35) and CSCCnon-LNM tissues (n = 40). **d** Statistical analysis revealed that the expressions of IL-10, Sp1 and CCL1 were higher in CSCCLNM tissues than in CSCCnon-LNM tissues. Clinical relevance was analysed by the Pearson’s coefficient test, and a significant correlation between IL-10 and Sp1+ lymphatic vessel (**e** *r* = 0.4257, *P* < 0.001), IL-10 and CCL1 + lymphatic vessel (**f** *r* = 0.3527, *P* < 0.001) was observed. TCGA data showed that IL-10 (**g** *P* = 0.0324), Sp1 (**h** *P* = 0.0296) and CCL1 (**i** *P* = 0.0436) overexpression correlated with poor prognosis in CSCC patients. *P < 0.05 by log-rank test. **j** Illustrative model showing the underlying mechanism of LVEM formation in the hypoxic TME

We then attempted to translate these findings to human CSCC patients. We observed that the number of hypoxic TAMs and LV density were significantly higher in CSCC^LNM^ tissues (*n* = 35) as compared with CSCC^non-LNM^ tissues (*n* = 40) (*P* < 0.05) (Fig. 6a and b). CSCC^LNM^ tissues showed significantly higher numbers of hypoxic TAMs proximal to LVs relative to CSCC^non-LNM^ tissues (*P* < 0.05) (Fig. 6c). Additionally, CSCC^LNM^ tissues displayed significantly higher expression of IL-10, Sp1, and CCL1 as compared with levels in CSCC^non-LNM^ tissues (*P* < 0.05) (Fig. 6d). Analysis of clinical relevance according to Pearson’s coefficient test indicated a significant correlation between IL-10 and Sp1^+^ LVs (*r* = 0.4257, *P* < 0.001) (Fig. 6e) and IL-10 and CCL1^+^ LVs (*r* = 0.3527, *P* < 0.001) (Fig. 6f). To evaluate the relevance of our observations to clinical prognosis, we analysed public data from The Cancer Genome Atlas (TCGA), which revealed that increased levels of IL-10, Sp1, and CCL1 correlate with poor prognosis in CSCC patients (*P* = 0.0324, *P* = 0.0296, and *P* = 0.0436, respectively) (Fig. 6g–i), further suggesting that IL-10, Sp1, and CCL1 play oncogenic roles in the metastatic progression of CSCC.

Collectively, our findings indicated that TAMs in hypoxic niches secrete large amounts of IL-10 to activate LECs in the surrounding TME. The activated Sp1^high^ LECs promote transactivation of CCL1, facilitating the recruitment of TAMs and tumour cells and forming a positive feedback loop to strengthen the LVEM (Fig. 6j). These results unveiled the underlying mechanism of LVEM formation and provide a strategy for inhibiting lymphatic metastasis and improving the prognosis of CSCC patients.

## Discussion

Tumour heterogeneity in the hypoxic TME is a potent driver of tumour progression and metastasis [5]. LVs in the hypoxic TME show remarkable plasticity and heterogeneity, reflecting their functional specialization [6]. Therefore, therapeutic strategies that non-selectively target the entire LV population are ineffective and can potentially lead to cancer progression [29]. In the present study, we discovered a novel lymphatic pattern and determined its clinical significance in CSCC. We found that the LVEM in hypoxic milieu provided an important pathway for lymphatic metastasis, by which activated LECs recruited tumour cells in a hypoxic TAM-dependent manner. Interestingly, CSCC^non-LNM^ is largely devoid of this LVEM, which might explain its metastatic delay and lower potential for metastasis. Moreover, the highly aggressive LVEM appears to be essential to tumour cell dissemination and might substantially contribute to the biological differences between CSCC^LNM^ and CSCC^non-LNM^ tissues. Thus, we provided mechanistic and clinical insight into the role of the LVEM in lymphatic metastasis.

In our previous study, we reported preferential accumulation of TAMs in hypoxic areas of tumours and their secretion of cytokines promoting the formation of pro-metastatic tumour niches [23]. Additionally, we found that in the hypoxic zones of a primary tumour site, a subset of TAMs adjacent to LVs constructed a novel lymphatic metastasis-promoting pattern. The general understanding of how TAMs influence lymphangiogenesis focuses on their production and release of growth factors and metalloproteases [30]. Nevertheless, the mechanisms by which a subset of TAMs adjacent to LVs participate in the formation of lymphatic conduits within the hypoxic TME, as well as their cooperation to facilitate tumour cell intravasation into LVs, remain unidentified. A recently identified mechanism suggests that podoplanin-expressing TAMs engage β1 integrins during the recruitment and adhesion of these cells to galectin-8-expressing lymphatics [31]. Once in the perilymphatic space, TAMs promote endothelial cell remodelling to enhance lymphangiogenesis and metastasis. In the present study, we identified and characterized a subset of hypoxic TAMs that localized proximal to tumour lymphatics and were relevant to lymphoinvasion. Both findings emphasize a TAM subset that specifically interacts with LVs to promote neo-lymphatic formation and the migration of tumour cells through the lymphatic system. Our study highlights the unique aggressive nature of the lymphatic metastasis-promoting pattern, which might emerge as a major characteristic to discriminate between CSCC^LNM^ and CSCC^non-LNM^ patients.

Hypoxic TAMs form a favourable niche for LVEM formation through IL-10 upregulation. CM collected from LECs treated with IL-10 stimulated tumour cell chemotaxis to an even greater extent than CM obtained from untreated LECs. Our data revealed that hypoxic TAMs but not tumour cells were the predominant source of IL-10. IL-10 stimulation also leads to a further upregulation of IL-10 in macrophages, which is an autocrine amplification cascade incited by IL-10 [32]. IL-10 is a multifunctional cytokine involved in anti-inflammatory and immunoregulatory effects [33]. In addition to its wide-ranging immunomodulatory functions, IL-10 also regulates lymphangiogenesis via macrophages [34]. In the present study, our data indicated that IL-10 was a potential mediator of cross-talk between TAMs and LECs in hypoxic zones, with this activity involved in mediating LVEM formation. In these zones, IL-10 is also an important determinant of alternative M2 activation and sustainment of tumour cell proliferation [35]. Higher lymphatic activity is mainly regarded as an increased opportunity for tumour cells to access the lymphatic system [36]. Blockade of IL-10 signalling resulted in abrogation of TAM-H-mediated LEC activation, and the observed increase in tumour cell chemotaxis to inflamed LECs was completely abolished by depleting IL-10R. However, IL-10 did not have a direct effect on tumour cell migration. Previous reports indicate that CSCC patients exhibiting upregulated levels of serum IL-10 showed poorer response to antitumour therapy relative to patients with low serum IL-10 levels [37]. The results of our present study revealed that hypoxic TAMs exhibiting elevated IL-10 expression formed a favourable niche for lymphangiogenesis and LVEM formation and promoted metastatic progression of CSCC, which might explain the poor therapeutic response of these patients.

We then examined the regulatory mechanisms of IL-10-mediated LVEM in CSCC^LNM^ tissues, finding that the niches formed by IL-10-activated LECs provided a constant source of paracrine CCL1 for TAM infiltration, which in turn further strengthened the LVEM. Chemokines were recently implicated in organ-specific metastasis of tumour cells [38], and human CCL1 is a potent monocyte chemoattractant that binds to and exclusively activates CCR8 [39]. In the present study, adding an anti-CCL1 neutralization antibody to IL-10-activated LEC CM or silencing CCR8 on receptor cells using siRNA or a CCR8 antagonist significantly impaired cell migration, indicating that the CCL1–CCR8 axis mediates most of the chemotactic response to LEC CM. CCR8 plays a rather unique role in regulating the immune response [40] and is preferentially expressed by activated T helper-type 2 cells and tumour cells [15], which mediates their recruitment to the lymphatics. Moreover, the CCL1–CCR8 axis also controls entry of other tumour-infiltrating lymphocytes into LNs for microenvironment remodelling [41]. For tumours to metastasize, tumour cells must gain enhanced migratory capacity, and the TME must be remodelled [42]. CCL1 produced by LECs mediates TAM migration towards LECs during LVEM formation while also promoting tumour cell entry into LNs by increasing tumour cell motility [43]. These results suggest that metastatic tumour cells are responsive to the chemotactic signals from lymphatic endothelium. Moreover, chemokines derived from the endothelium are essential for integrin-mediated adhesion and transendothelial cell migration [44]. These data not only emphasize the indispensable role of LVEM in LNM but also indicate that CCL1 might represent an attractive alternative therapeutic target to interfere with CSCC metastasis.

In this scenario, there is a pressing need to identify more specific and convenient markers to distinguish metastasis-associated LVs for precision treatment. Our analysis of the CCL1 promoter region identified Sp1 as a transcription regulator in LECs that contributes to CSCC metastasis. Furthermore, LVEM^high^ CSCC tissues displayed a higher Sp1 and CCL1 level relative to that in LVEM^low^ cases. Sp1 is a highly regulated transcription factor involved in regulating a large number of genes that contribute to the “hallmarks of cancer” [45]. Sp1 also involved in upregulating the lymphangiogenic genes, such as VEGF-C, VEGFR-3 to promote lymphangiogenesis [46, 47]. Additionally, Sp1 is essential for early embryonic development but dispensable for cell growth and differentiation [48], with Sp1 expression changing during development and varying in different cell types [49]. Previous studies reported that Sp1 expression correlated with dismal patient outcome in various cancer types, including CSCC [50]. Notably, accumulating evidence shows that Sp1 plays a critical role in the inflammatory signalling that mediates cancer-stroma cross-talk [51]. Therefore, investigating the actions associated with aberrant activation of Sp1 is important to understanding tumour progression. In this study, Sp1 was suggested as a downstream TF of the IL-10/STAT3 pathway. Moreover, we found that metastasis-associated LVs could play an active role in tumour metastasis, which was at least partly due to elevated expression of Sp1. Importantly, we confirmed that blockade of Sp1 or CCL1 prevented TAM proximity to LVs, thereby reducing tumour lymphangiogenesis and metastatic dissemination. This suggests that identification of the Sp1^high^ LVEM subset would have a profound impact on prognosis assessment. Our data revealed Sp1 as a key factor in a previously unexplored metastatic cascade in CSCC and promote further screening and potential development of Sp1-specific inhibitors in cancer therapy. Furthermore, analysis of Sp1^high^ LVs in a tumour could be an indication for complete LN resection or adjuvant therapy. The prognostic values of LVs previously denoted by conventional markers, such as LYVE-1 or D2-40, are often different or even opposing in different studies [52, 53]. Indeed, single expression of LYVE-1 cannot precisely predict LNM states [54]. The present study provides a molecular definition for metastasis-associated LVs that suggests anti-Sp1 therapy as potentially beneficial for LVEM^high^ CSCC patients through its inhibition of LVEM formation. A larger cohort of patients is warranted to further explore this idea.

Lymphatic metastasis is the principal reason for the poor survival rates of CSCC patients. A detailed understanding of how LVs contribute to further metastasis is crucial to better comprehend the biological complexity of lymphatic metastasis. In this study, we defined a lymphatic metastasis-promoting pattern characterized by a lymphatic skeleton surrounded by TAMs, with this pattern prevalent in CSCC^LNM^ tissue and actively involved in lymphatic metastasis. Moreover, M2 macrophages were selectively recruited by Sp1^high^ LECs to form this unique metastasis-promoting pattern. Identification of the LVEM and its regulatory mechanisms not only offers novel targets for the development of anti-metastasis therapy but also provides a basis for the selection of specific cohorts of patients who might benefit from certain molecular-targeted drugs.

## Supplementary Information

Below is the link to the electronic supplementary material.

Supplementary file1 (DOCX 1436 KB)
