# Correction: A novel lymphatic pattern promotes metastasis of cervical cancer in a hypoxic tumour-associated macrophage-dependent manner

**DOI:** 10.1007/s10456-022-09854-5

**Published:** 2022-09-10

**Authors:** Xiao-Jing Chen, Wen-Fei Wei, Zi-Ci Wang, Nisha Wang, Chu-Hong Guo, Chen-Fei Zhou, Luo-Jiao Liang, Sha Wu, Li Liang, Wei Wang

**Affiliations:** 1 Department of Obstetrics and Gynecology, The First Affiliated Hospital of Guangzhou Medical University, 151 Yanjiang Road, Yuexiu District, Guangzhou, 510120 People’s Republic of China; 2 Department of Immunology/Guangdong Provincial Key Laboratory of Proteomics, School of Basic Medical Sciences, Southern Medical University, 1838 Guangzhou Avenue North, Baiyun District, Guangzhou, 510515 People’s Republic of China; 3 Department of Pathology, Nanfang Hospital, Southern Medical University, 1838 Guangzhou Avenue North, Baiyun District, Guangzhou, 510515 People’s Republic of China; 4 Department of Biochemistry and Molecular Biology, School of Basic Medical Sciences, Southern Medical University, 1838 Guangzhou Avenue North, Baiyun District, Guangzhou, 510515 People’s Republic of China

## Correction to: Angiogenesis (2021) 24:549–565 10.1007/s10456-020-09766-2

The authors would like to correct Figs. 2 and 3, as errors were introduced in the preparation of the figures for publication. The authors declare that these corrections do not change the results or conclusions of this paper. The authors have provided the corrected version of Figs. 2 and 3 here. The authors also would like to correct the description of statistics in the Methodology Statistics section as “The results are expressed as the mean value ± SEM and were interpreted by the *t*-test or one-way analysis of variance (ANOVA)” because of the incomplete description in the original paper.

The revised Fig. 2 and Fig. 3 are as follows:

**Fig. 2:**
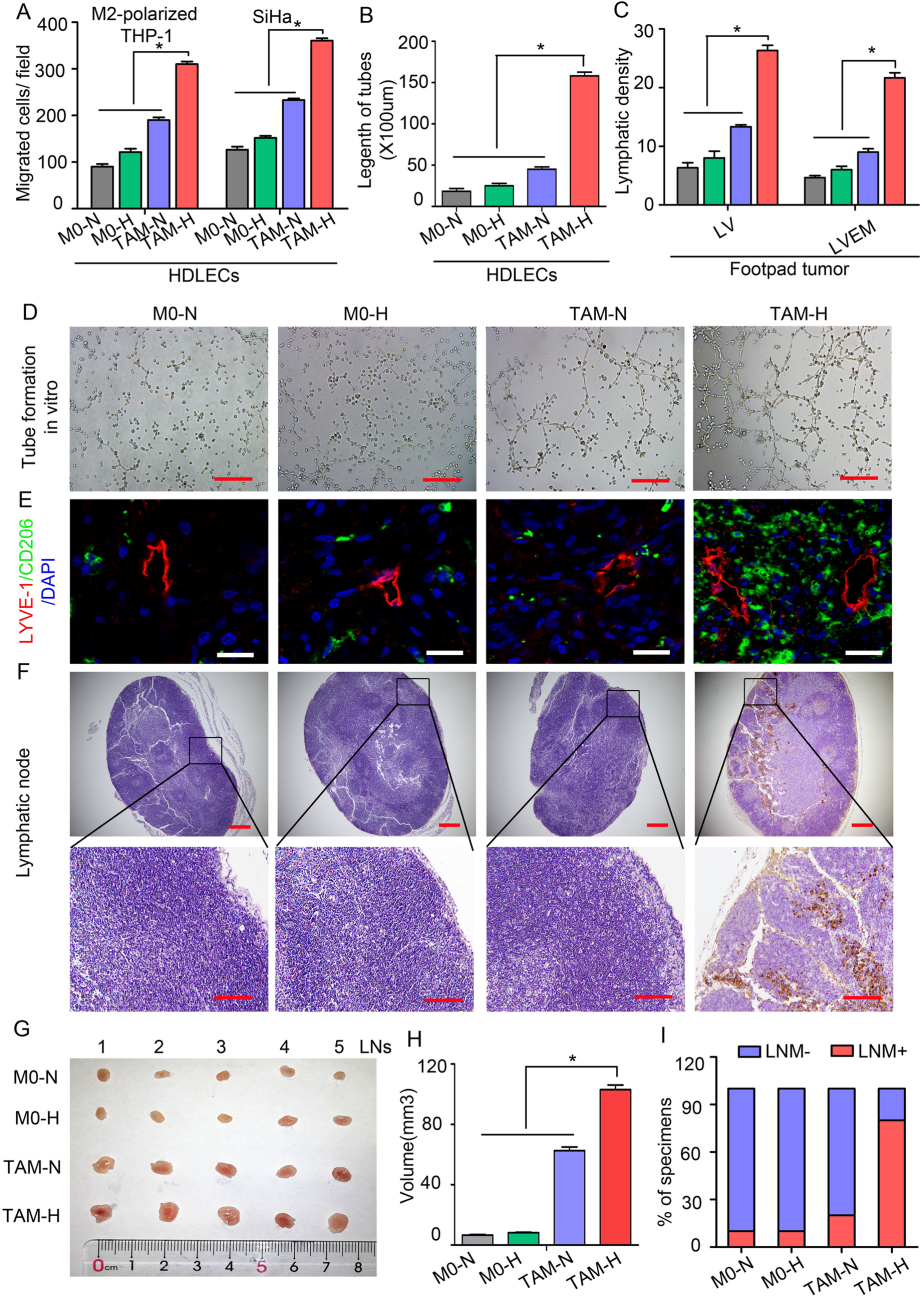
Hypoxic TAMs promote lymphangiogenesis and LVEM formation. **a** The function of CM from different macrophage-treated HDLECs on M2-polarized THP-1 macrophages and tumour cells (SiHa) was detected by transwell array in vitro. **b** Statistical analysis showing the length of tube formation in vitro. Average length of tubes per field were calculated. **c** Statistical analysis showing the expression of LV and LVEM in mouse footpad tumour. **d** Representative micrographs showing tube formation assay in vitro of HDLECs pretreated with different macrophage CM for 48 h. Scale bar, 50 μm. **e–i** Popliteal lymphatic metastasis model was established in female C57BL/6 mice by inoculating the footpad with TC-1 cells (5 × 10^6^). When footpad tumour size reached 50 mm^3^, macrophage supernatants of different treatment conditions (10 μL) were then injected into the center of the tumours (*n* = 5/group, repeated twice) for 2 weeks daily. After 2 weeks of induction, primary tumours reached a comparable size of ~ 150 mm^3^, and then footpad tumours and popliteal LNs were collected for study. **e** Representative images of LYVE-1^+^ lymphatic vessel (red), CD206^+^ TAMs (green) and DAPI (blue) fluorescence staining in footpad tumour. Images are shown at × 400 magnification (Scale bar, 50 μm). **f** IHC Staining of CK7 in popliteal LNs. Representative micrographs are shown (Scale bar, 100 μm). Metastasis-positive LNs were identified by staining for epithelial marker CK7. **g** Photos of mouse popliteal LNs in different macrophage CM-primed tumour (*n* = 5/group). **h** Statistical analysis showing the volume (mm^3^) of the LNs. **i** The ratio of metastasis-positive to total dissected popliteal LNs from mice treated with different macrophage supernatants. Error bars represent the mean ± SD of three independent experiments. *P < 0.05. *N* Normoxia, *H* Hypoxia

**Fig. 3:**
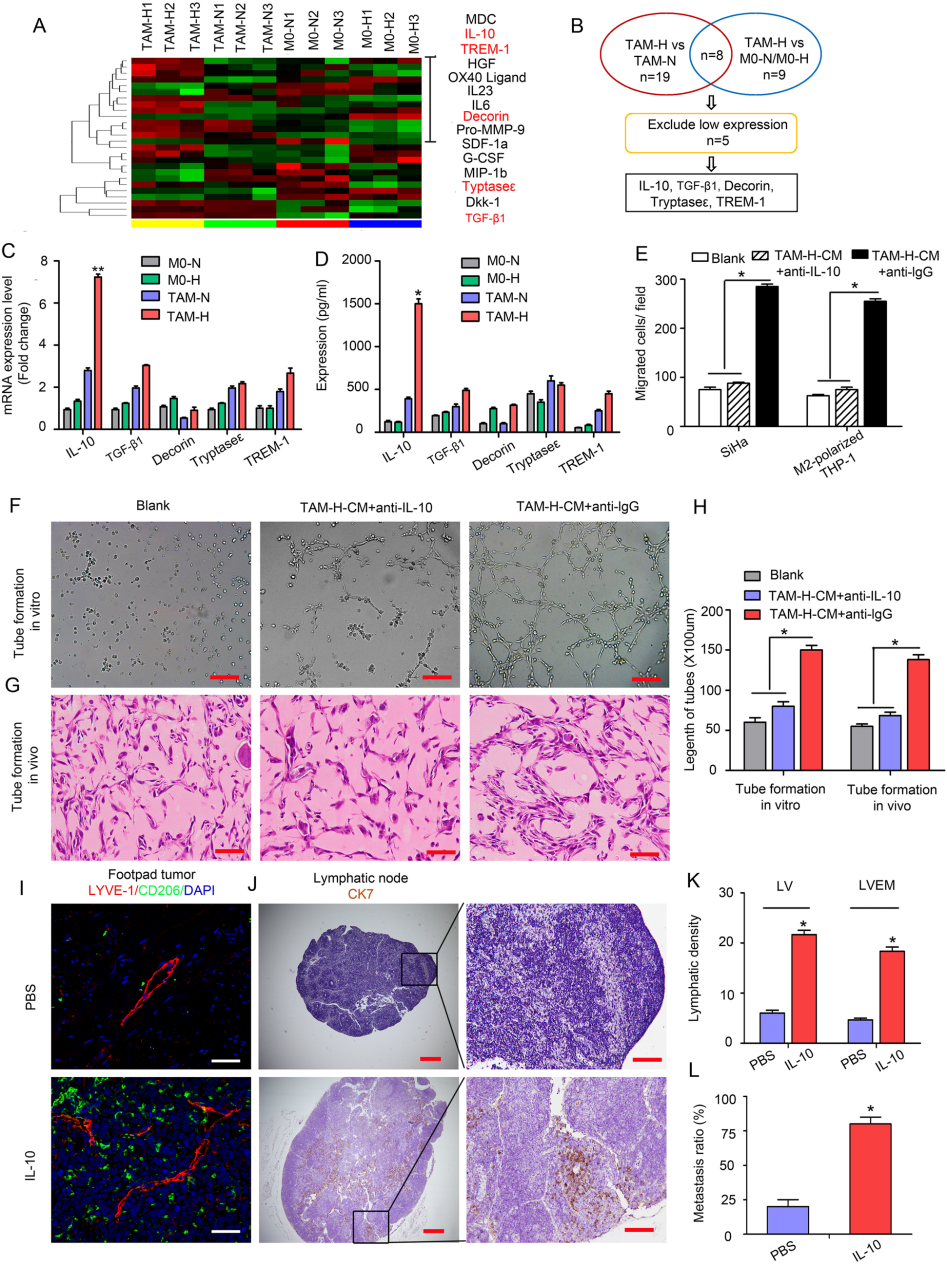
IL-10 derived from hypoxic TAMs is required to maintain LVEM. **a** The different cytokines expression profiles among M0-N, M0-H, TAM-N and TAM-H were analysed by cytokine array (RayBio GSM-CAA-4000). **b** Screening and analysis of the differentially expressed cytokines. **c** The expressions of the five significant cytokines were analysed by qRT-PCR. **d** The secretions of the five significant cytokines were analysed by ELISA. **e** The migration effects of hypoxic TAMs-treated HDLECs on tumour cells (SiHa) and M2-polarized THP-1 macrophages were analysed by transwell assay in vitro. “Blank” represents the medium group. **f** Representative micrographs showing the tube formation in vitro (Scale bar, 50 μm). **g** Representative images showing the tube formation in vivo (Scale bar, 100 μm). **h** Statistical analysis showing the length of tube formation. Average length of tubes per field were calculated. **i–l** Popliteal lymphatic metastasis model was established in female C57BL/6 mice by inoculating the footpad with TC-1 cells (5 × 10^6^). When footpad tumour size reached 50 mm^3^, IL-10 (50 ng/mL) or PBS was then injected into the centre of the tumours (*n* = 5/group, repeated twice) for 2 weeks daily. After 2 weeks of induction, primary tumours reached a comparable size of ~ 150 mm^3^, and then footpad tumours and popliteal LNs were collected for study. **i** Representative images of LYVE-1^+^ lymphatic vessel (red), CD206^+^ TAMs (green) and DAPI (blue) fluorescence staining in footpad tumour. **j** Metastasis-positive LNs were identified by IHC staining for epithelial marker CK7. **k** Statistical analysis showing the expression of peritumoural LV and LVEM in footpad tumour. **l** Statistical analysis showing the ratio of LNM. Error bars represent the mean ± SD of three independent experiments. **P < 0.01
