# Supplementary material for: A novel lymphatic pattern promotes metastasis of cervical cancer in a hypoxic tumour-associated macrophage-dependent manner
Source: Angiogenesis. 2021 Jan 23;24(3):549–65. doi: 10.1007/s10456-020-09766-2 (PMC8292274; doi:10.1007/s10456-020-09766-2)
Supplement: Supplementary file 1 — Supplementary file1 (DOCX 1436 KB) [file 10456_2020_9766_MOESM1_ESM.docx]

**Supplemental Figures and Figure legends**

**
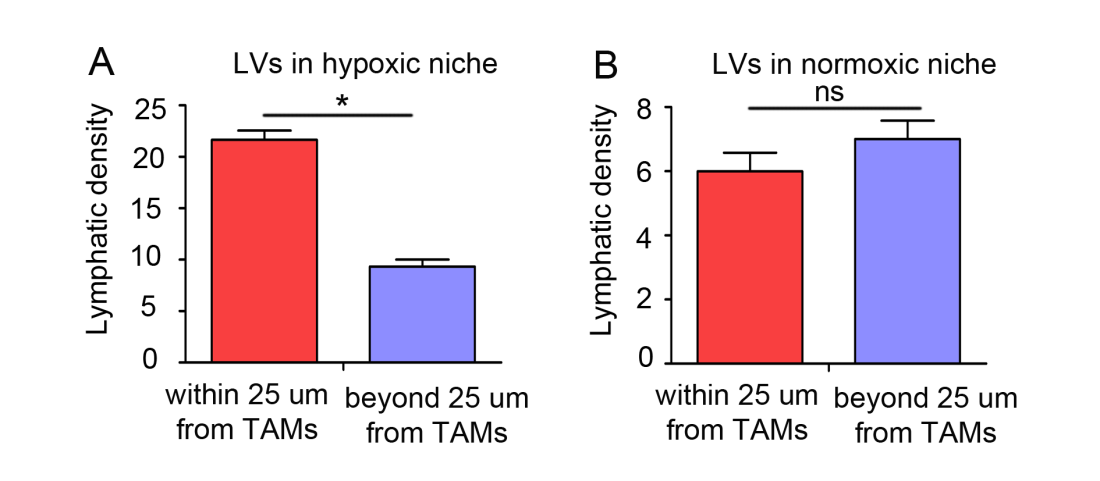
**

**Supplemental Fig. 1 Lymphatic vessels (LVs) surrounding TAMs in the hypoxic niche are involved in lymphangiogenesis.**

(a-b) Statistical analysis of the lymphatic density surrounding TAMs (within or beyond a distance of 25 μm from TAMs) in hypoxic (a) and normoxic (b) regions. *, *P*<0.05. ns, no significance.

**
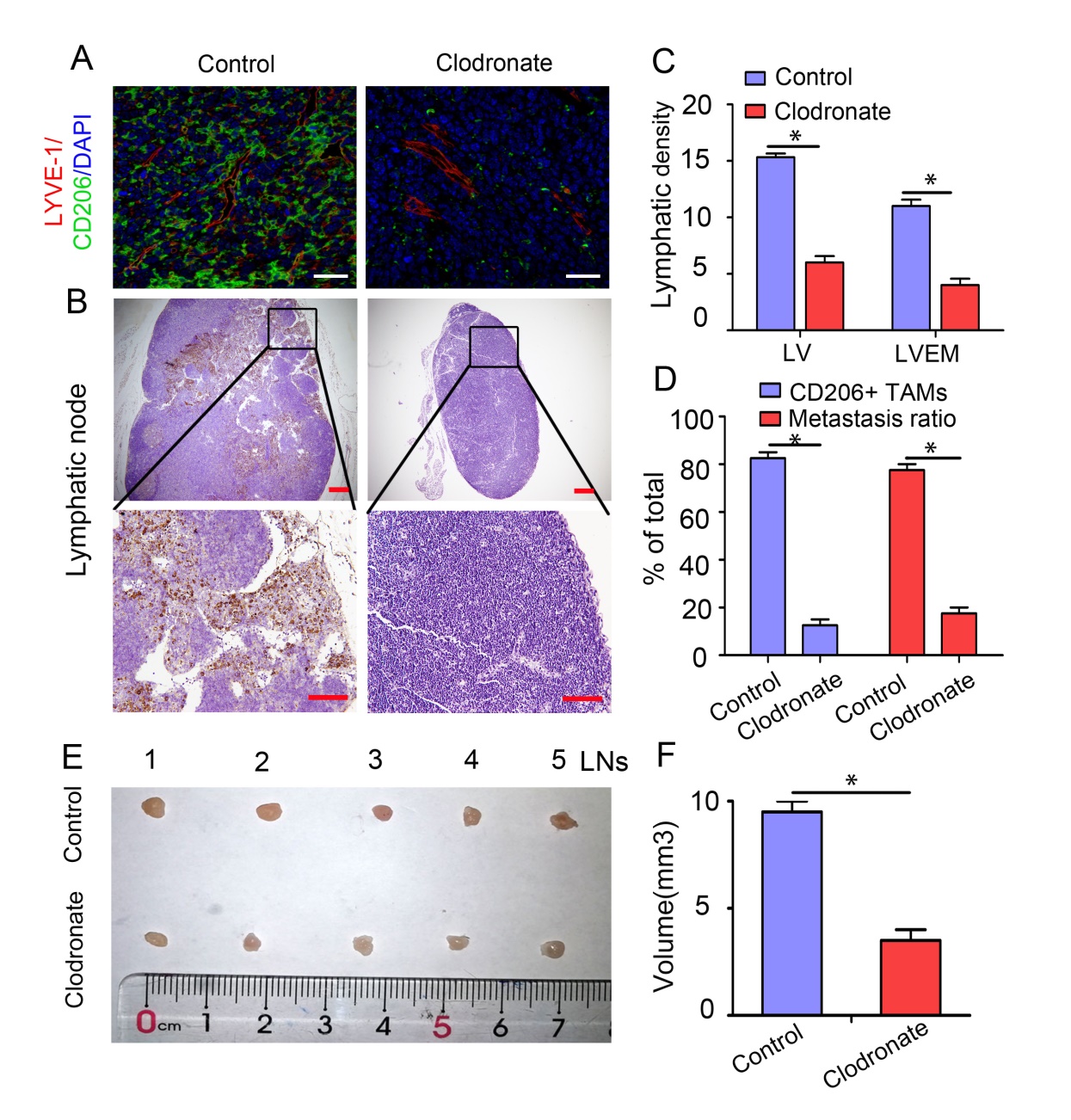
**

**Supplemental Fig. 2 TAMs facilitate lymph node metastasis (LNM).**

(a) Representative images of LYVE-1^+^ lymphatic vessel (red), CD206^+^ TAMs (green) and DAPI (blue) fluorescence staining in footpad tumour of mice receiving clodronate or control liposome. Scale bar, 100 μm. (b) IHC Staining of CK7 in popliteal LNs from mice treated with different liposomes (Scale bar, 100 μm). (c) Statistical analysis showing the expression of of peritumoural LV and LVEM in footpad tumour. (d) Statistical analysis showing the quantification of CD206^+^ TAMs and the ratio of LNM. (e) Photos of mouse popliteal LNs in clodronate or control liposome-primed tumour. (f) Statistical analysis showing the volume (mm^3^) of the popliteal LNs. Error bars represent the mean ± SD of three independent experiments. *, *P*<0.05.

**
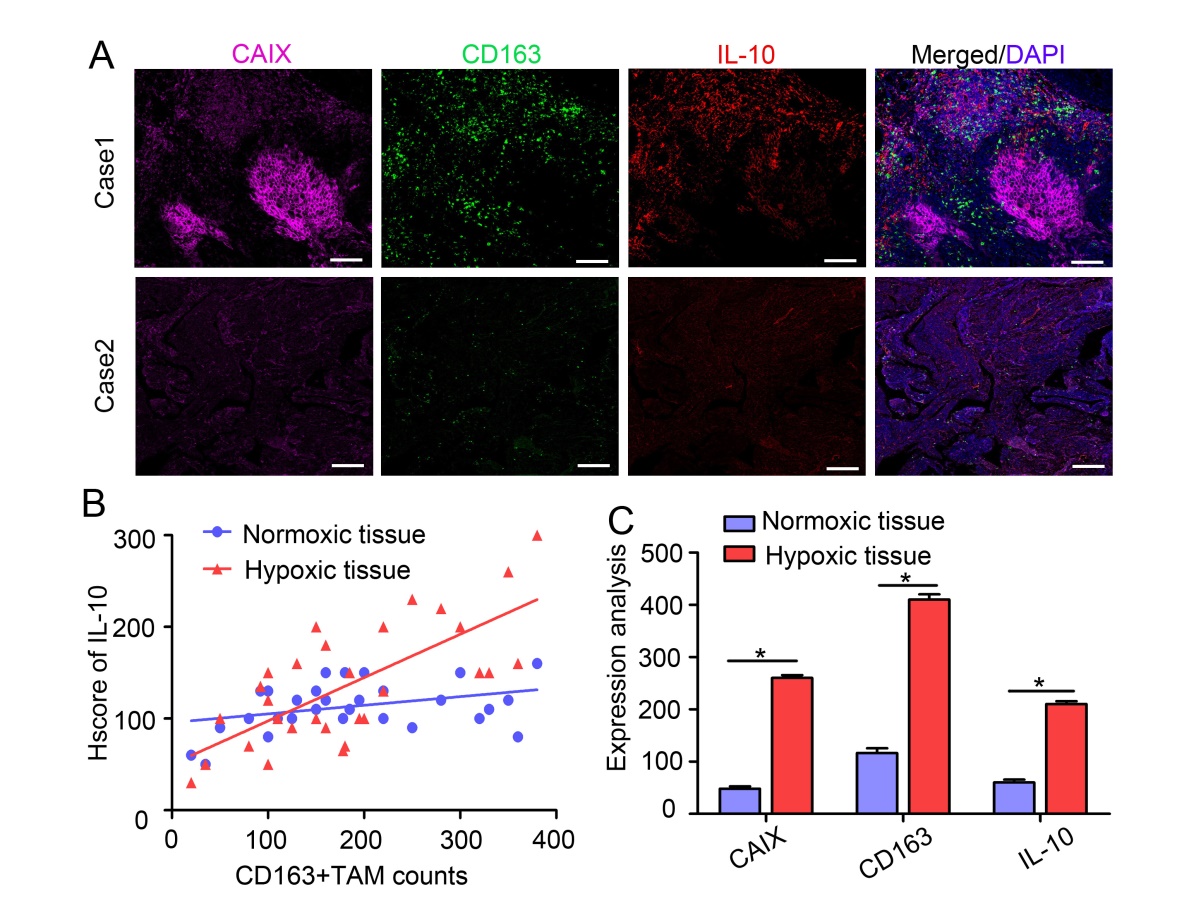
**

**Supplemental Fig. 3 The expression analysis of IL-10 in CSCC tissue.**

(a) Representative images of CAIX (purple), CD163^+^ TAMs (green), IL-10 (red) and DAPI (blue) fluorescence staining in CSCC tissues. Scale bar, 100 μm. (b) The correlation between CD163^+^ TAMs and IL-10 was statistically analysed by Pearsons’ coefficient test (red line, r=0.5178, *P*<0.0001; blue line, r=0.1253, *P*=0.0550). (c) Statistical analysis showing the expression of CAIX, CD163 and IL-10 in hypoxic and normoxic regions of CSCC tissues. Error bars represent the mean ± SD of three independent experiments.*, *P*<0.05

**
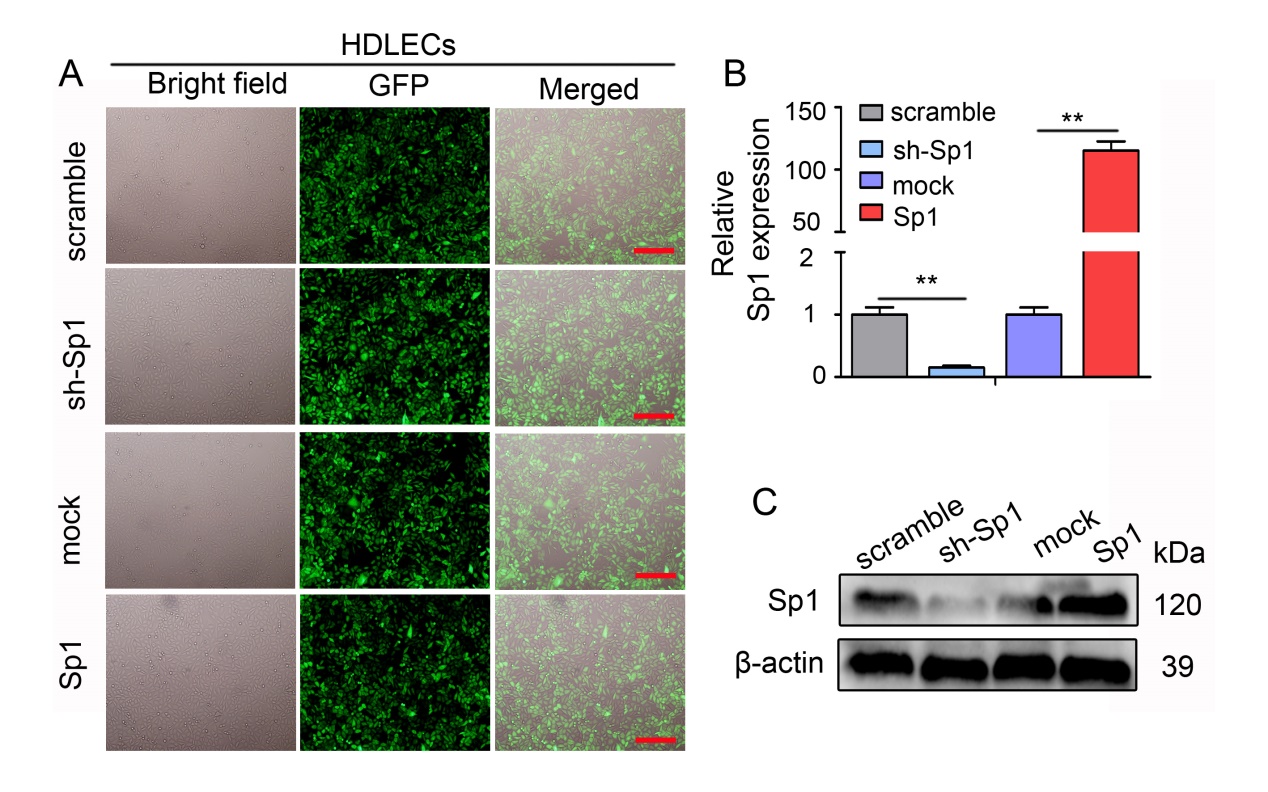
**

**Supplemental Fig. 4 HDLECs with stable overexpression or silencing of Sp1 were established.**

(a) GFP images of HDLECs with silencing, stable overexpression of Sp1 and their respective negative control (scramble and mock) (Scale bar, 100 μm). (b) qRT-PCR showing the RNA expression level of Sp1 in HDLECs with stable overexpression or silencing of Sp1. (c) Western blot showing the protein expression level of Sp1 in HDLECs with stable overexpression or silencing of Sp1. Error bars represent the mean ± SD of three independent experiments. *, *P*<0.05

**
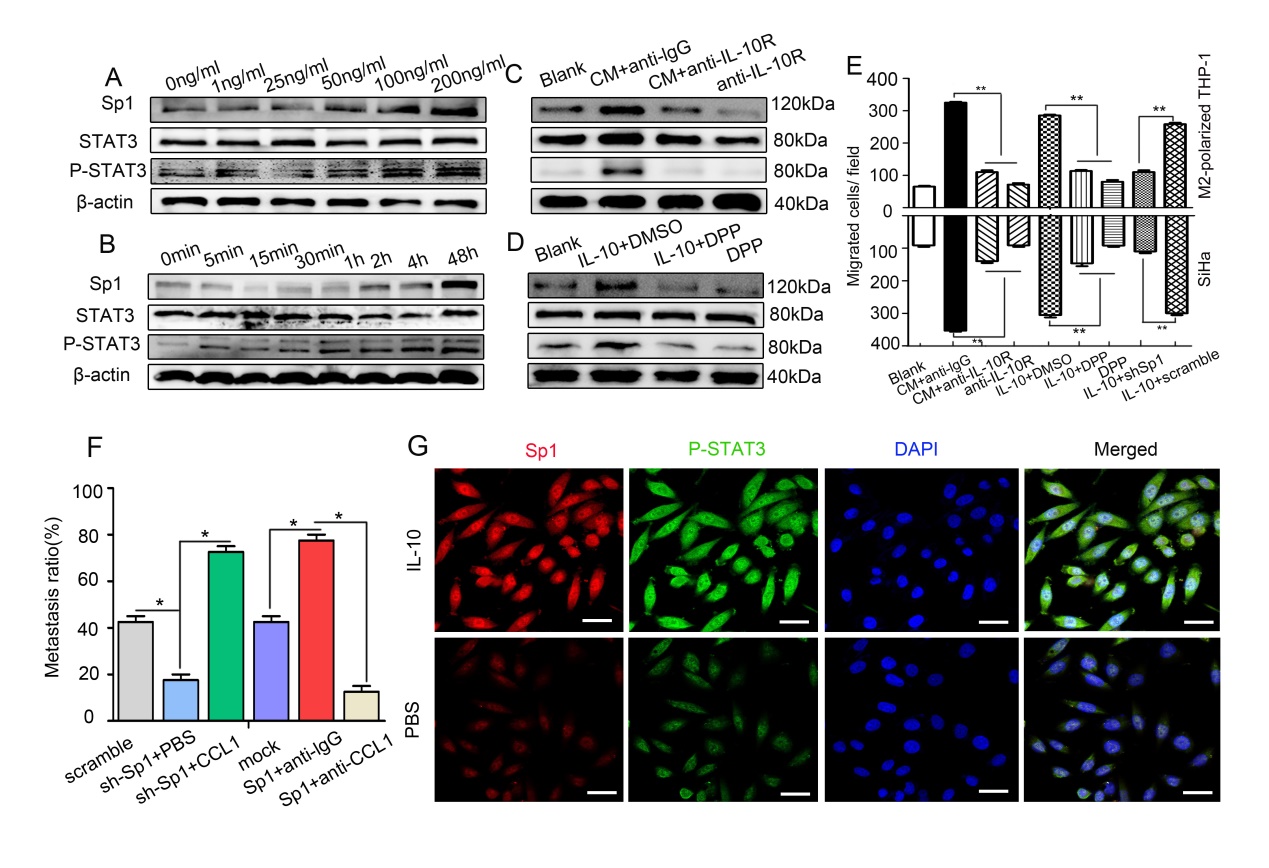
**

**Supplemental Fig. 5 IL-10-STAT3 signalling pathway involved in LVEM formation.**

(a-b) Western blot revealed that incubation of HDLECs with IL-10 led to increased expression of Sp1, via activation of phosphorylation STAT3 signalling. (c-d) After inhibition of STAT3 signal (5, 15-diphenyl-porphine (DPP), a potent inhibotor of STAT3) or adding anti-IL-10R neutralization antibody, Sp1 expression on HDLECs was obviously decreased. (e) The migration effects of M2-polarized THP-1 macrophages and tumour cells were detected by transwell array respectively. “Blank” represents the medium group. (f) Popliteal lymphatic metastasis model was established in female C57BL/6 mice by inoculating the footpad with TC-1 cells (5×10^6^). When footpad tumour size reached 50 mm^3^, HDLEC supernatants of stable overexpression, silencing of Sp1, and their respective negative control cells (10 μL) were then injected into the center of the tumours (*n* = 5/group, repeated twice) for 2 weeks daily. CCL1(50ng/ml)/PBS or anti-CCL1/ anti-lgG neutralization antibody was respectively adding. After 2 weeks of induction, primary tumours reached a comparable size of ~ 150 mm^3^, and then popliteal LNs were collected for study. Metastasis-positive LNs were identified by staining for epithelial marker CK7. Statistical analysis showing the ratio of LNM. (g) Immunofluorescence showing the expression of Sp1 and phosphorylated STAT3 in HDLECs incubated with IL-10 (Scale bar, 100 μm). Error bars represent the mean ± SD of three independent experiments. **, *P*<0.01.

Supplemental Table 1. Primers for real-time RT-PCR.

| Gene | PCR primers(5’-3’) | |
| --- | --- | --- |
|  | Sense primers | Antisense primers |
| β-actin | CCATCAATGACCCCTTCATTGACC | GAAGGCCATGCCAGTGAGCTTCC |
| Sp1 | GTGGAGGCAACATCATTGCTG | GCCACTGGTACATTGGTCACAT |
| IL10 | AGCCTTATCGGAAATGATCCAGT | GGCCTTGTAGACACCTTGGT |
| Decorin | ACCTCTCGTGAAGTTGGAAAGG | CCCAGAGTTTTTCAGTGGGTTG |
| Tryptase | CATTTCTGCGGAGGTTCTCTC | CACCACGATCCTGTTCAAAGA |
| TREM-1 | TGCTGTGCGTGTTCTTTGTCT | CCTCCACTAGGTCATACCTTTCT |
| CCL1 | CTCATTTGCGGAGCAAGAGAT | GCCTCTGAACCCATCCAACTG |
| CCL2 | CAGCCAGATGCAATCAATGCC | TGGAATCCTGAACCCACTTCT |
| CCL3 | AGTTCTCTGCATCACTTGCTG | CGGCTTCGCTTGGTTAGGAA |
| CCL5 | CCAGCAGTCGTCTTTGTCAC | CTCTGGGTTGGCACACACTT |
| CCL7 | TGTCCTTTCTCAGAGTGGTTCT | TGCTTCCATAGGGACATCATA |
| CCL8 | TGGAGAGCTACACAAGAATCACC | TGGTCCAGATGCTTCATGGAA |
| CSF1 | TGGCGAGCAGGAGTATCAC | AGGTCTCCATCTGACTGTCAAT |
| CCL19 | TACATCGTGAGGAACTTCCACT | CTGGATGATGCGTTCTACCCA |
| CCL21 | GTTGCCTCAAGTACAGCCAAA | AGAACAGGATAGCTGGGATGG |
| CXCL8 | ACTGAGAGTGATTGAGAGTGGAC | AACCCTCTGCACCCAGTTTTC |
| CXCL9 | CCAGTAGTGAGAAAGGGTCGC | AGGGCTTGGGGCAAATTGTT |
| CXCL10 | GTGGCATTCAAGGAGTACCTC | TGATGGCCTTCGATTCTGGATT |
| CXCL11 | GACGCTGTCTTTGCATAGGC | GGATTTAGGCATCGTTGTCCTTT |
| CXCL12 | ATTCTCAACACTCCAAACTGTGC | ACTTTAGCTTCGGGTCAATGC |
